# Local and Global Rigidification Upon Antibody Affinity Maturation

**DOI:** 10.3389/fmolb.2020.00182

**Published:** 2020-08-07

**Authors:** Monica L. Fernández-Quintero, Johannes R. Loeffler, Lisa M. Bacher, Franz Waibl, Clarissa A. Seidler, Klaus R. Liedl

**Affiliations:** Center for Molecular Biosciences Innsbruck, Institute of General, Inorganic and Theoretical Chemistry, University of Innsbruck, Innsbruck, Austria

**Keywords:** antibodies, CDR-H3 loop, affinity maturation, rigidification, localizing plasticity, kinetics, Markov-state models

## Abstract

During the affinity maturation process the immune system produces antibodies with higher specificity and activity through various rounds of somatic hypermutations in response to an antigen. Elucidating the affinity maturation process is fundamental in understanding immunity and in the development of biotherapeutics. Therefore, we analyzed 10 pairs of antibody fragments differing in their specificity and in distinct stages of affinity maturation using metadynamics in combination with molecular dynamics (MD) simulations. We investigated differences in flexibility of the CDR-H3 loop and global changes in plasticity upon affinity maturation. Among all antibody pairs we observed a substantial rigidification in flexibility and plasticity reflected in a substantial decrease of conformational diversity. To visualize and characterize these findings we used Markov-states models to reconstruct the kinetics of CDR-H3 loop dynamics and for the first time provide a method to define and localize surface plasticity upon affinity maturation.

## Graphical Abstract

Visualization of the local and global rigidification as a consequence of affinity maturation.

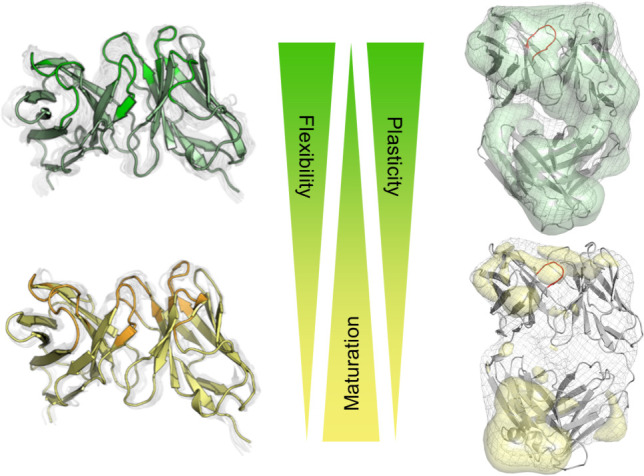

## Introduction

Since the identification of antibodies in the 19th century, the rise and importance of monoclonal antibodies as biotherapeutics over the past 30 years has been extraordinary (Carter, 2006, 2011; Reichert, 2017; Kaplon and Reichert, 2019). Antibodies are composed of two polypepide chains, called V_H_ and V_L_ (Edelman, 1973). Each chain consists of a variable and a constant region. The variable domain contains six hypervariable loops, referred to as the complementarity determining regions (CDRs), which shape the antigen-binding site, the paratope (Nguyen et al., 2017). The specificity of an antibody is mainly influenced by the CDR loops and therefore characterization of the paratope is essential for understanding the function of the antibody (James et al., 2003). Five of the six CDR loops, except the CDR-H3 loop, can adopt a limited number of main-chain conformations and have been classified into canonical structures according to their length and sequence composition (Chothia and Lesk, 1987; Al-Lazikani et al., 1997). The highest variability in sequence, length and structure of an antibody can be observed in the CDRs, especially in the CDR-H3 loop, while antibody frameworks are fairly well conserved (∼150 human germline framework sequences). The CDR-H3 loop plays a central role in antigen recognition and has on average the highest counts of contacts with antigens (Marks and Deane, 2017; Regep et al., 2017). Structure prediction of the CDR-H3 loop due to its exceptional diversity of both structure and sequence and the ability to adopt various different conformations during V(D)J recombination and somatic hypermutation remains challenging (Bassing et al., 2002; Market and Papavasiliou, 2003; Clark et al., 2006; Burkovitz et al., 2014). Additionally, the CDR-H3 loop length and structure have an effect on the antigen-binding patterns of the CDR loops and influence the specificity of the paratope for target antigens. Thus, to elucidate the role of B cells in adaptive immunity and the evolution of antibodies binding specific antigens, the understanding of the affinity maturation process and its effects on the CDR loops, especially on the CDR-H3 loop, are crucial. The binding site of polyreactive monoclonal antibodies, which bind with low affinity to various structurally unrelated antigens, has been discussed to be significantly more flexible compared to matured antibodies (Zhou et al., 2007; Gunti and Notkins, 2015). Depending on the antigen present, polyreactive antibodies show a broader and shallower free energy surface, reflected in various different binding site conformations and higher conformational diversity of the paratope (Schmidt et al., 2013; Fernández-Quintero et al., 2019b). Especially the CDR-H3 loop substantially influences the shape of the paratope and thus plays a central role in antigen-binding. The correlation between rigidification and enhanced specificity has been discussed in terms of conformational selection (Ma et al., 1999; Tsai et al., 1999). Antibody-antigen binding can be interpreted to follow the paradigm of conformational selection. This implies an ensemble of pre-existing conformations with different probabilities, in which the binding-competent state is selected (Tsai et al., 1999; Csermely et al., 2010). Repeated exposure to the same antigen leads to mutations in the sequences which can result in a rigidification of the antigen binding site. Various studies focused on the effects of affinity maturation on the CDRs suggesting that structural rigidification and less conformational diversity are a consequence of affinity maturation (Wedemayer et al., 1997; Manivel et al., 2000; Yin et al., 2001, 2003; Li et al., 2003; Thielges et al., 2008; Adhikary et al., 2012, 2015; Schmidt et al., 2013; Jeliazkov et al., 2018). Additionally, 3-pulse photon echo peak shift (3PEPS) spectroscopy has been used to quantify antibody dynamics on the femto-to nanosecond timescale. A direct comparison between naïve with mature antibodies showed that mature antibodies can be characterized by a higher rigidity, reflected in smaller motions and conformational changes than naïve antibodies (Jimenez et al., 2003; Adhikary et al., 2012, 2015). Additionally, numerous MD studies investigated and showed the rigidification of the CDR-H3 loop as a consequence of affinity maturation (Thorpe and Brooks, 2007; Wong et al., 2011; Schmidt et al., 2013). Recently, it has been reported that antibody CDR-H3 loops does not result in a rigidification (Jeliazkov et al., 2018), but it has also been shown that on a significantly longer timescale the CDR-H3 loop rigidifies upon affinity maturation (Fernández-Quintero et al., 2019b). Thus, the affinity maturation process represents a direct connection between an enhanced specificity and rigidification. However, rigidification is only one of numerous biophysical mechanisms responsible for the increase in affinity (Jeliazkov et al., 2018).

In this study, we focus on characterizing the conformational diversity of the CDR-H3 loop including transition probabilities and changes in surface plasticity of 10 pairs of antibody fragments upon affinity maturation. We based our investigation on strong experimental structural information and compared naïve (before exposure to an antigen) and matured (after repeated exposure to an antigen) antibodies crystallized with and without the presence of the antigen.

## Materials and Methods

A previously published method characterizing the CDR-H3 loop ensemble upon antigen-binding in solution (Fernández-Quintero et al., 2019a, b, 2020a,b,c) was used to investigate the conformational diversity of CDR-H3 loop upon affinity maturation. Experimental structural information was available for all considered antibody fragments (Fabs and Fvs).

**FIGURE 1:**
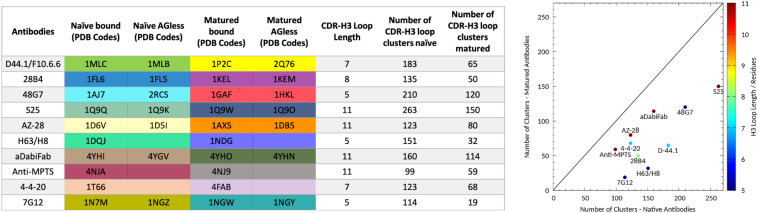
Overview of all antibody fragments analyzed with the available PDB accession codes crystallized with and without antigen and in the naive and matured state. Additionally, the resulting number of clusters of the CDR-H3 loop of the naïve and matured antibody fragments by using the same distance cut-off of 1.2 Å is shown on the left and visualized on the right. The plot on the right shows the number of clusters of the naïve antibody against the number of clusters of the matured antibody color-coded according to their loop length.

To avoid repetition, we only discuss three pairs of antibody fragments upon affinity maturation in detail, while the results for the other antibodies are summarized in Figure 1. The structural changes upon affinity maturation for all ten antibody pairs are visualized and described in more detail in the Supplementary Material (Supplementary Tables 4–12). This 10 pairs of antibodies undergoing affinity maturation were chosen as they have been part of previous work considering the effects of affinity maturation on antibody flexibility (Wedemayer et al., 1997; Yin et al., 2001; Jimenez et al., 2003; Li et al., 2003, 2015; Zimmermann et al., 2006; Thorpe and Brooks, 2007; Thielges et al., 2008; Babor and Kortemme, 2009; Wong et al., 2011; Schmidt et al., 2013; Willis et al., 2013; Adhikary et al., 2015; Schiele et al., 2015; Jeliazkov et al., 2018; Fernández-Quintero et al., 2019b).

The first affinity maturation pair analyzed is the D44.1 (naïve) and the F10.6.6 (matured) anti-lysozyme antibody Fab crystallized with and without the antigen lysozyme (Braden et al., 1994). Both antibody Fabs are murine monoclonal antibodies which are related in sequence and structure as they originate from the same germline gene rearrangement. The available PDB accession codes of the naïve and matured antibody fragments crystallized with and without antigen are 1MLC, 1MLB, and 2Q76, 1P2C, respectively (Braden et al., 1994; Cauerhff et al., 2004; Acierno et al., 2007). D44.1 Fab differs from the affinity matured variant F10.6.6 in 20 mutations, seven of them located in the CDR loops. Due to the occurring mutations, structural changes yielded in a stabilized V_H_–V_L_ interface with an increase in the affinity toward the antigen. A significant increase in the number of non-covalent bonds between the antibody and the antigen from the naïve complex 93 to the matured complex 129 as well as closer and stronger bonds were observed. The second studied affinity maturation pair is the 28B4 Fab, which catalyzes a periodate-dependent oxidation of sulfide to sulfoxide, whereby the hapten (1-[N-4′-nitrobenzyl-N-4′-carboxybutylamino] methylphosphonic acid) was generated to mimic the transition state of this reaction (Hsieh-Wilson et al., 1996; Yin et al., 2001). The available experimental structures are germline Fabs crystallized with and without the hapten present (PDB codes: 1FL6 and 1FL5) and the respective affinity matured Fab variants (1KEL and 1KEM). Nine mutations, two in the V_L_ and seven in the V_H_ were introduced during affinity maturation. Three of these mutated residues of the matured antibody, Asn35H, Lys56H, and Trp101H directly interact with the hapten. A decrease in flexibility and changes in the binding geometry of the antigen due to these mutations led to an increased complementarity and affinity between the antibody and the hapten. The third pair of affinity maturation antibodies is the esterolytic antibody 48G7, which catalyzes an ester and carbonate hydrolysis reaction, whereby the hapten 5-(para-nitrophenyl phosphonate)-pentanoic acid portrays the transition state (Wedemayer et al., 1997). Available crystal structures that were used as starting structures for MD simulations are the germline Fab fragment in complex with and without the antigen present (PDB codes: 1AJ7 and 2RCS) as well as the corresponding affinity matured structures (PDB codes: 1GAF and 1HKL). During the process of affinity maturation, nine mutations were introduced, three in the V_L_ and six in the V_H_.

The starting structures for simulations were prepared in MOE (Molecular Operating Environment, Chemical Computing Group, version 2018.01) using the Protonate3D tool (Labute, 2009; Molecular Operating Environment [MOE], 2018). To neutralize the charges we used the uniform background charge (Roe and Cheatham, 2013; Hub et al., 2014; Case et al., 2016). Using the tleap tool of the AmberTools16 (Roe and Cheatham, 2013; Case et al., 2016) package, the crystal structures were soaked with cubic water boxes of TIP3P water molecules with a minimum wall distance of 10 Å to the protein (Jorgensen et al., 1983). For all crystal structures parameters of the AMBER force field 14SB were used (Maier et al., 2015). The antibody fragments were carefully equilibrated using a multistep equilibration protocol (Wallnoefer et al., 2011).

### Metadynamics Simulations

To enhance the sampling of the conformational space well-tempered metadynamics (Barducci et al., 2008, 2011; Biswas et al., 2018) simulations were performed in GROMACS (Pronk et al., 2013; Abraham et al., 2015) with the PLUMED 2 implementation (Tribello et al., 2014). We used a linear combination of sine and cosine of the ψ torsion angles of the CDR-H3 and CDR-L3 loop as collective variables, calculated with functions MATHEVAL and COMBINE implemented in PLUMED 2 (Tribello et al., 2014). As discussed previously the ψ torsion angle captures conformational transitions comprehensively (Ramachandran et al., 1963; Wood and Hirst, 2005; Fernández-Quintero et al., 2019b). The decision to include the CDR-L3 loop ψ torsion angles is based on the structural correlation of the CDR-L3 and CDR-H3 loop and the observed improved sampling efficiency (James and Tawfik, 2005). The simulations were performed at 300 K in an NpT ensemble. We used a Gaussian height of 10.0 kcal/mol. Gaussian deposition occurred every 1,000 steps and a biasfactor of 10 was used. 1 μs metadynamics simulations were performed for each available antibody fragment crystal structure. The resulting trajectories were clustered by using the average linkage hierarchical clustering algorithm in CPPTRAJ (Shao et al., 2007; Roe and Cheatham, 2013) with a distance cut-off criterion of 1.2 Å resulting in a large number of clusters. The cluster representatives for the antibody fragments were equilibrated and simulated for 100 ns using the AMBER18 (Case et al., 2016) simulation package.

### Molecular Dynamics Simulations

Molecular dynamics simulations were performed in an NpT ensemble using pmemd.cuda (Salomon-Ferrer et al., 2013). Bonds involving hydrogen atoms were restrained by applying the SHAKE algorithm (Miyamoto and Kollman, 1992), allowing a time step of 2.0 fs. Atmospheric pressure of the system was preserved by weak coupling to an external bath using the Berendsen algorithm (Berendsen et al., 1984). The Langevin thermostat (Adelman and Doll, 1976) was used to maintain the temperature during simulations at 300 K.

For the obtained trajectories a tICA was performed using the python library PyEMMA 2 employing a lag time of 10 ns (Scherer et al., 2015). Thereby, a dimensionality reduction is obtained by transforming the trajectories into an intuitive measure, e.g., backbone torsions, which represent the slowest coordinates of the system (Pérez-Hernández and Noé, 2016; Wu and Noé, 2017). To construct the tICA we chose as input variables the backbone torsions of the CDR-H3 loop. The first two tICs (time-lagged independent components) describe the two slowest components of the CDR-H3 loop movements. Thermodynamics and kinetics were calculated with a Markov-state model (Chodera and Noé, 2014) by using PyEMMA 2, which uses the k-means clustering algorithm (Likas et al., 2003) to define microstates and the PCCA + clustering algorithm to coarse grain the microstates to macrostates. PCCA + is a spectral clustering method, which discretizes the sampled conformational space based on the eigenvectors of the transition matrix (Röblitz and Weber, 2013). Markov-state models allow to identify significant structural changes during the simulation and reconstruct thermodynamics and kinetics. The sampling efficiency and the reliability of the Markov-state model (e.g., defining optimal feature mappings) can be evaluated with the Chapman–Kolmogorov test (Karush, 1961; Miroshin, 2016), by using the variational approach for Markov processes (Wu and Noé, 2017) and by taking into account the fraction of states used, as the network states must be fully connected to calculate probabilities of transitions and the relative equilibrium probabilities. To build the Markov-state model we used the backbone torsions of the CDR-H3 loop, defined 150 microstates using the k-means clustering algorithm and applied a lag time of 10 ns.

### Characterization of Surface Plasticity

Conformational plasticity of proteins has been shown to play key role in molecular mechanisms such as catalytic activity, biomolecular recognition and allosteric regulation (Daberdaku and Ferrari, 2018; Jespersen et al., 2019). Differences of the antibody surface were calculated by using the average surface of the simulation and the respective standard deviations of each frame. To visualize the differences in plasticity upon affinity maturation, we calculated the per-voxel average and standard deviation of the reconstructed grid. The standard deviation is useful to highlight regions that are sometimes occupied by the protein and sometimes solvent-accessible. Flexible regions are characterized by large volumes with high standard deviation. However, the resulting grid is difficult to interpret because even very rigid regions can have a few partially occupied voxels. To emphasize regions with large structural differences, we applied a Gauss filter to smooth the average and the standard deviation grid. To test our method, we used the anti-MPTS Fv, previously analyzed to address the influence of the affinity maturation on the CDR-H3 loop (Fernández-Quintero et al., 2019b), to compare experimentally measured plasticity via 3PEPS spectroscopy (Adhikary et al., 2015) with our calculated plasticity. 3PEPS has been successfully used to characterize protein dynamics such as side chain rotations and loop rearrangements (Oh et al., 2011; Adhikary et al., 2012). In line with the experiment we observe a decrease in plasticity and flexibility for the further matured 8B10 Fv (Supplementary Figure S1).

## Results

Various studies have discussed the effect of affinity maturation on structural and dynamic properties (James and Tawfik, 2003; Cauerhff et al., 2004; Schmidt et al., 2013; Adhikary et al., 2015; Jeliazkov et al., 2018; Shehata et al., 2019).

We analyzed 10 pairs of antibody fragments supported by strong experimental structural information upon affinity maturation and a summary of the resulting CDR-H3 loop flexibilities of the respective antibody pairs is illustrated in Figure 1. On the left the PDB accession codes, the CDR-H3 loop lengths and the resulting numbers of clusters by using the same distance cut-off criterion of 1.2 Å, are displayed for all studied antibody fragments. On the right the number of CDR-H3 loop clusters of the naïve and matured antibody fragments are plotted against each other to visualize the substantial rigidification upon affinity maturation. The clustering also been performed using different cut-off criteria to see if the results presented in Figure 1 are stable under variation of the cut-off and in all cases the native antibodies reveal a higher number of clusters, indicating a higher flexibility of the CDR-H3 loop before maturation.

**FIGURE 2:**
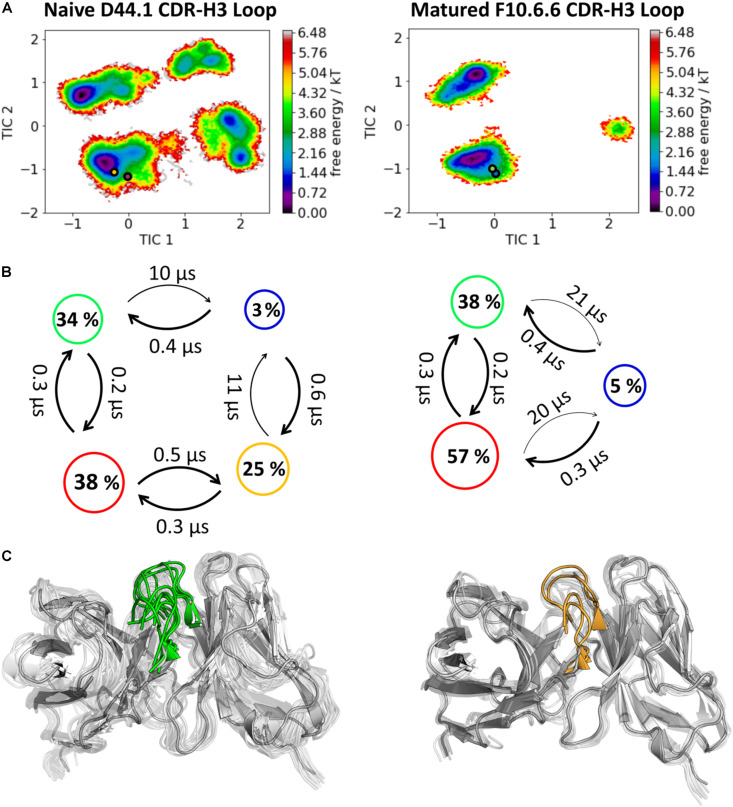
Kinetic, thermodynamic and structural analyses of the CDR-H3 loop ensemble in solution. **(A)** Free energy surface of the naive D44.1 and the matured F10.6.6 Fab in the same coordinate system, including the respective X-ray structures crystallized with and without antigen. The orange and green dots show the bound X-ray structures of the naïve and the matured Fab, respectively, while magenta and blue display the X-ray structures crystallized without antigen. **(B)** Transition timescales between the different macrostates orientated according to the tICA space including the state probabilities. **(C)** Substantial decrease of the conformational ensemble of the D44.1 and F10.6.6 antibody upon affinity maturation.

**FIGURE 3:**
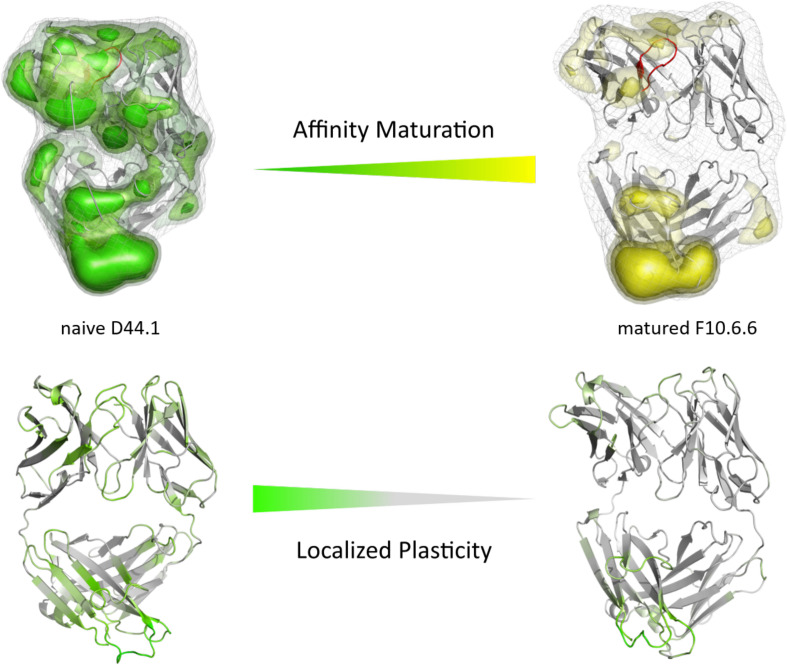
Global **(top)** and localized **(bottom)** surface plasticity of the naive D44.1 and matured F10.6.6 antibody Fab mapped onto a representative ensemble structure. **(Top)** Global plasticity of the naïve and the matured D44.1 and F10.6.6 antibody fragments, highlighting the CDR-H3 loop in red. The intensity of the colors reflects the regions with higher plasticity and thus allows localization. **(Bottom)** Localized plasticity for both the naïve and the matured D44.1 and F10.6.6 antibody fragment Fab, respectively.

As described in the “Materials and Methods” section, we used the cluster representatives as starting structures for each 100 ns MD simulations to be able to reconstruct and characterize thermodynamics and kinetics. Figure 2A displays the resulting free energy surface of 18.3 μs of the naïve D44.1 Fab and 6.5 μs of the matured F10.6.6 Fab in the same coordinate system. Upon affinity maturation a substantial rigidification of the CDR-H3 loop dynamics combined with a population shift toward the global minimum in solution could be observed. Figure 3C shows the resulting CDR-H3 loop ensemble in solution color-coded according to Figure 1 and emphasizes the significant decrease in conformational diversity. Supplementary Figure S2 illustrates the 2D-RMSD plots (based on the Cα coordinates) and the B-factors of the CDR-H3 loop. In line with the decrease in conformational space of the CDR-H3 loop, which can be seen in Figure 3, the rigidification of the CDR-H3 loop is reflected in both the 2D-RMSD and the B-factors. Besides, characterizing flexibility by the resulting number of clusters, RMSF or 2D-RMSD plots, we developed a method to analyze and localize surface plasticity of antibody fragments (Figure 3). Figure 3 shows the projection of the calculated plasticity of the naïve and the matured antibody Fab onto a representative ensemble structure. The intensity of the colors reflects regions with higher plasticity. As surface plasticity is an essential aspect of biomolecular recognition, we find that characterization of protein plasticity allows a better shape-based interpretation of the antigen binding site, compared to other flexibility measures such as RMSD and B-factors. Upon affinity maturation we observe a significant decrease in surface plasticity. Also, the CDR-H3 loop reveals substantially less plasticity in the matured F10.6.6 Fab. This observation is in line with the decrease in conformational diversity, in particular of the CDR-H3 loop. The 2D-RMSD plots of both the paratope and the whole variable fragment are illustrated in the Supplementary Figure S3 and clearly show a global rigidification upon affinity maturation.

**FIGURE 4:**
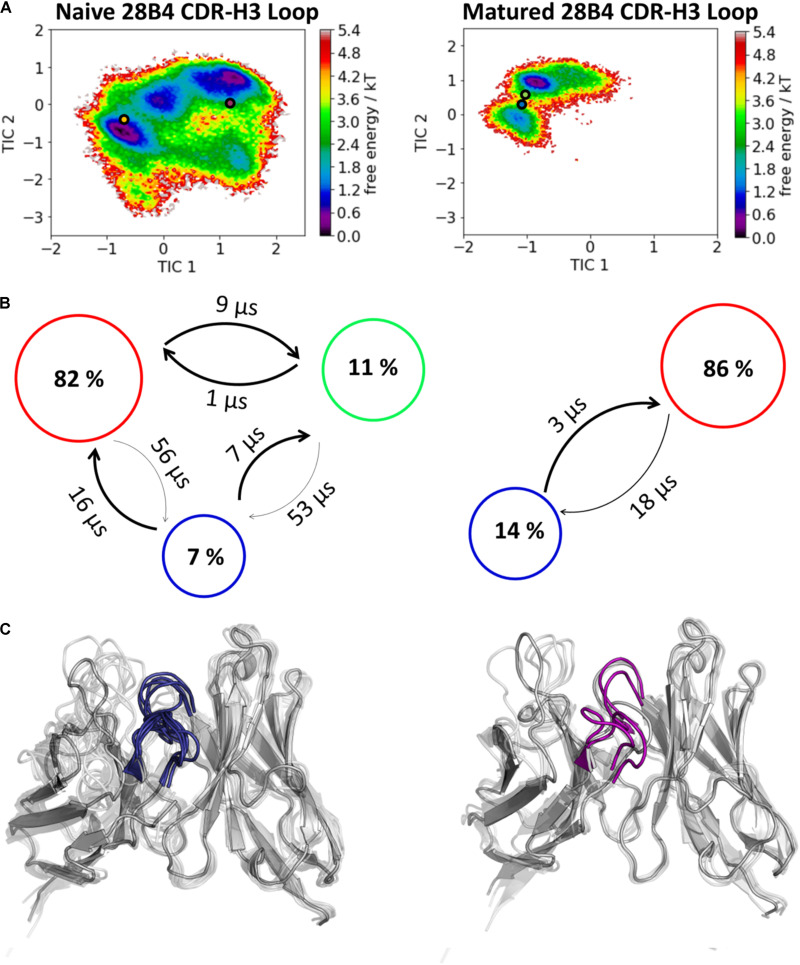
Kinetic, thermodynamic and structural analyses of the CDR-H3 loop ensemble in solution upon affinity maturation. **(A)** Free energy surface of the naive and matured 28B4 Fab in the same coordinate system, including the respective X-ray structures crystallized with and without antigen. The orange and green dots show the bound X-ray structures of the naïve and the matured Fab, respectively, while magenta and blue display the X-ray structures crystallized without antigen. **(B)** Transition timescales between the different macrostates orientated according to the tICA space including the state probabilities. **(C)** Substantial decrease of the conformational ensemble of the 28B4 antibody upon affinity maturation.

**FIGURE 5:**
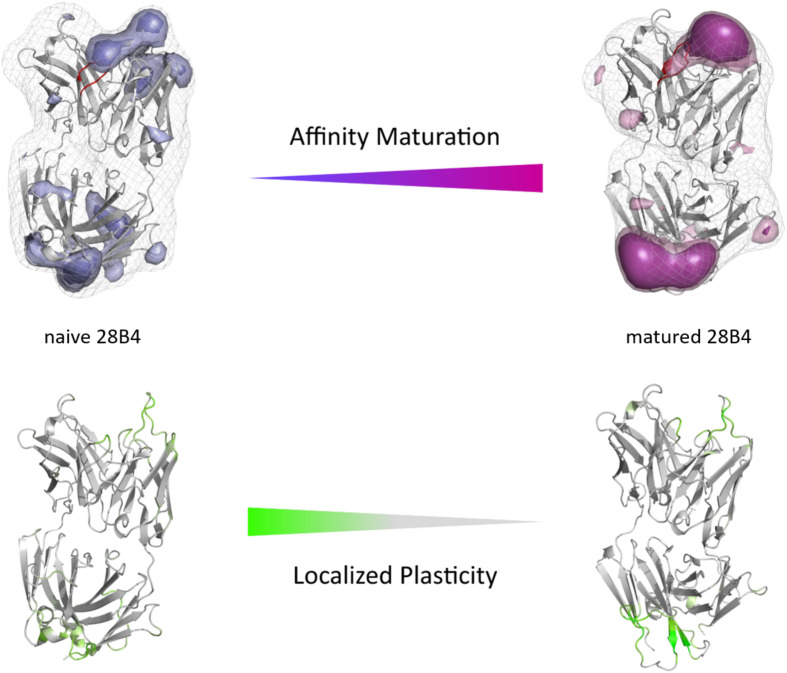
Global **(top)** and localized **(bottom)** surface plasticity of the naive and matured 28B4 antibody Fab mapped onto a representative ensemble structure. **(Top)** Global plasticity of the naïve and the matured 28B4 antibody, highlighting the CDR-H3 loop in red. The intensity of the colors reflects the regions with higher plasticity and allows localization. **(Bottom)** Localized plasticity for both the naïve and the matured 28B4 antibody, respectively.

The second studied affinity maturation pair is the hapten-binding 28B4 antibody Fab. Figure 4A shows the resulting tICA plots of the resulting 13.5 μs trajectories of the naïve and 5.0 μs trajectories of matured 28B4 antibody in the same coordinate system. The available crystal structures are projected into the free energy landscape and color-coded respectively. The conformational ensemble of the CDR-H3 loop in solution reveals a substantial rigidification upon affinity maturation, reflected in a substantial decrease in conformational diversity (Figure 4). This significant rigidification of the CDR-H3 loop is also shown in Supplementary Figure S4. The 2D-RMSD plot clearly depict this decrease in flexibility upon affinity maturation. This finding is supported by the B-factors calculated for the CDR-H3 loop, as always higher values are obtained for the naïve antibodies. Figure 4C visualizes the substantial rigidification in the observed conformational diversity, which agrees with previous results. The effect of affinity maturation on the plasticity of the 28B4 antibody is visualized in Figure 5. Again, in line with the first analyzed pair we observe a decrease in plasticity upon affinity maturation, especially in the region of the CDR-H3 and CDR-L3 loop. This observation is confirmed by the 2D-RMSD plots of the paratope and the variable fragment illustrated in Supplementary Figure S5.

**FIGURE 6:**
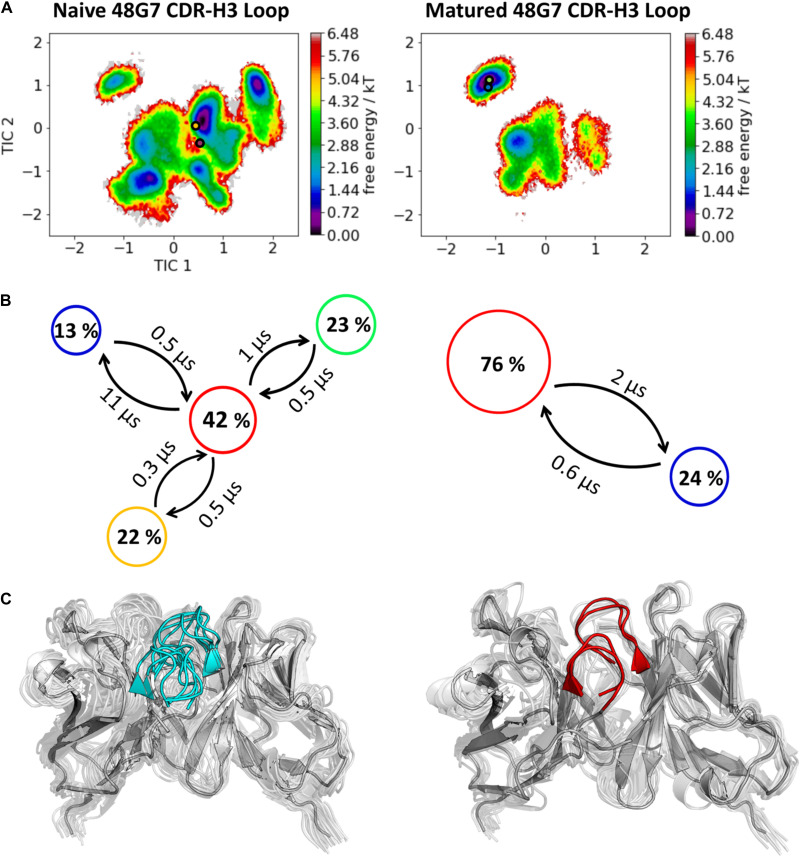
Kinetic, thermodynamic and structural analyses of the CDR-H3 loop ensemble in solution upon affinity maturation. **(A)** Free energy surface of the naive and matured 48G7 Fab in the same coordinate system, including the respective X-ray structures crystallized with and without antigen. The orange and green dots show the bound X-ray structures of the naïve and the matured Fab, respectively, while magenta and blue display the X-ray structures crystallized without antigen. **(B)** Transition timescales between the different macrostates orientated according to the tICA space including the state probabilities. **(C)** Substantial decrease of the conformational ensemble of the 48G7 antibody upon affinity maturation.

**FIGURE 7:**
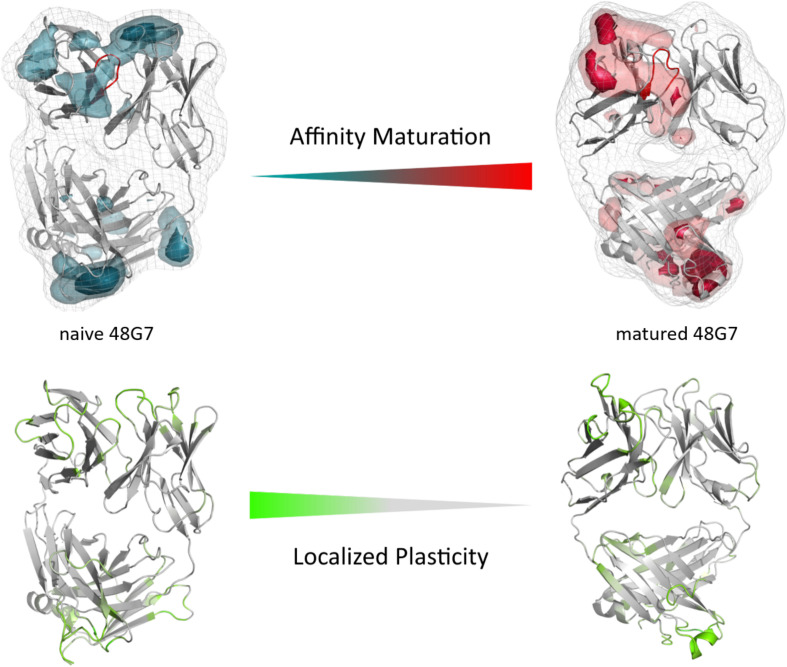
Global **(top)** and localized **(bottom)** surface plasticity of the naive and matured 4G87 antibody Fab mapped onto a representative ensemble structure. **(Top)** Global plasticity of the naïve and the matured 48G7 antibody, highlighting the CDR-H3 loop in red. The intensity of the colors reflects the regions with higher plasticity and allows localization. **(Bottom)** Localized plasticity for both the naïve and the matured 48G7 antibody, respectively.

The third in detail discussed affinity maturation pair is the 48G7 hapten binding antibody. Figure 6A reflects in agreement in with the substantial decrease in the number of CDR-H3 loop clusters (210 to 120), as a metric of quantifying flexibility, a substantial reduction in conformational space of the CDR-H3 loop. A representative conformational ensemble of the resulting 21 μs (naïve) and 12 μs (matured) trajectories revealing this significant decrease in conformational diversity is illustrated in Figure 6C. This finding is in line with localized flexibility metrics, such as the B-factors and the 2D-RMSD of the CDR-H3 loop shown in Supplementary Figure S6. Figure 6B illustrates the transition probabilities between the obtained macrostates for both the naive and the matured antibody fragment and shows the populations of the respective states. We clearly see that upon affinity maturation the dominant minimum in solution is shifted and the binding competent state becomes the most dominant state in solution (76%). Analysis of the resulting plasticity in Figure 7 displays significant reduction, especially in the CDR-H3 loop upon affinity maturation, which is highlighted by the localized plasticity in Figure 7 (bottom). 2D-RMSD plots for the paratope and the variable fragment of the 48G7 antibody are depicted in Supplementary Figure S7 and confirm this overall rigidification upon affinity maturation.

## Discussion

In this present study, we characterize the conformational diversity and the kinetic and thermodynamic properties of the CDR-H3 loop of 10 affinity maturation antibody pairs and present a method to visualize, localize and describe plasticity of antibodies upon affinity maturation. The affinity maturation process comprises the introduction of combinatorial mutations that increase the binding affinity of the antibody to the antigen and lead to a more effective immune response (French et al., 1989). Numerous structural studies, involving small molecules (haptens) were carried out comparing affinity-matured antibodies and their germline precursor binding to the same antigen (Alzari et al., 1990; Hsieh-Wilson et al., 1996; Chong et al., 1999; Yin et al., 2001; Mishra and Mariuzza, 2018). Thereby, somatic hypermutations in the CDR loops lead to a higher number of hydrogen bonds, electrostatic interactions, van der Waals contacts and an improved shape complementarity (Fernández-Quintero et al., 2019a, 2020b,c). Large conformational preorganization of the paratope in combination with a decrease in flexibility upon affinity maturation has been discussed to increase specificity for the target antigen while reducing the possibility of cross-reactivity with other antigens (Wedemayer et al., 1997; Manivel et al., 2000). Compared to affinity maturation studies focusing on haptens, structural affinity maturation of an antibody in response to a protein, i.e., hen egg white lysozyme, could not be attributed to a higher number of formed hydrogen bonds or salt bridges, but to an improved shape complementarity at the V_H_-binding interface accompanied by an increase of hydrophobic interactions (Braden et al., 1994; Li et al., 2003; DeKosky et al., 2016). In order to understand the mechanism of antigen-recognition, characterization of the thermodynamic and kinetics pathway of the affinity maturation process in combination with experimental structural information is crucial (Foote and Milstein, 1991, 1994; Milstein, 1991; Akiba and Tsumoto, 2015). Thus, the results presented in this study highlight that static structural information alone might not be sufficient to describe antibody binding properties as specificity and promiscuity (Akiba and Tsumoto, 2015; Fernández-Quintero et al., 2019b; Alba et al., 2020). Long timescale dynamics from enhanced and classic MD simulations complement experimental structural information with reliable estimations of flexibilities, state probabilities, binding mechanisms, and localization of plasticity. Figure 1 displays an overview of all studied affinity maturation antibody fragments including the resulting number of CDR-H3 loop clusters by using the same distance cut-off criterion, as a quantification of rigidification upon affinity maturation. We also investigated the stability of the results in dependence of the clustering cut-off and observed the same trend, that upon affinity maturation the flexibility of the CDR-H3 loop decreases substantially. Figures 2A,B show the free energy surface of the naïve D44.1 and the matured F10.6.6 Fab in the same tICA coordinate system and reveal a substantial decrease in conformational space of the CDR-H3 loop. The naïve D44.1 Fab displays a broader free energy landscape, compared to the deeper and narrower minima observed for the matured F10.6.6 Fab. Besides, we identified that even without the antigen present within the pre-existing ensemble of conformations, the binding competent state lies in the dominant minimum in solution. This indicates that the D44.1 Fab follows the paradigm of conformational selection. The two highest populated states of the CDR-H3 loop in solution of the naïve D44.1 Fab are the dominant conformations of the matured F10.6.6 Fab. The binding competent state in the naïve antibody becomes the highest populated state upon affinity maturation (38 → 58% state population). Figure 3C illustrates the conformational ensemble of the CDR-H3 loop and emphasizes the substantial reduction in conformational diversity upon affinity maturation. This substantial rigidification upon affinity maturation is supported by the 2D-RMSD plots and the B-factors illustrated in Supplementary Figures S2, S3. Figure 3 visualizes and localizes differences and regions with high plasticity. We did not only observe an overall decrease in plasticity, but we could also identify a substantial reduction in the CDR-H3 loop surface plasticity. Supplementary Figure S8 depicts the localized surface plasticity for the CDR-H3 loop for all in detail investigated antibody fragments and Supplementary Table 13 summarizes the overall reduction in plasticity upon affinity maturation. Figures 4A,B illustrate in line with the observations of the D44.1.1/F10.6.6 affinity maturation study, a substantial rigidification of the CDR-H3 loop conformational space of the 28B4 Fab upon affinity maturation. Besides the substantial rigidification we identified that the dominant structure in solution was optimized to bind the antigen, while the Fab X-ray structure crystallized without antigen lies in a local shallow side-minimum, because of the distortion of the loop due to crystal contacts with the tail region of a symmetry mate Fab. The transition kinetics of the CDR-H3 loop for both the naïve and the matured Fab occur in the nano-to microsecond timescale. Additionally, we also observe a strong population shift upon affinity maturation. Again, the significantly reduced conformational ensemble is illustrated in Figure 4C and supports in line with all other observations the rigidification upon affinity maturation. Figure 5 visualizes differences in plasticity of the 28B4 affinity maturation study and clearly shows in particular for the CDR-H3 and CDR-L3 loop a substantial decrease in surface plasticity. In agreement with these results Figures 6A,B show not only a decrease in conformational diversity of the CDR-H3 loop of the 48G7 Fab upon affinity maturation, but clearly reveals a population shift toward the dominant solution structure in the affinity matured Fab. Astonishingly, the dominant CDR-H3 loop conformation of the matured 48G7 Fab is present as a local shallow side-minimum in the free energy surface of the naïve 48G7 Fab. These findings supported the hypothesis that promiscuity might arise from numerous weakly populated conformations each of which is able to bind different binding partners (Zhou et al., 2007; Adhikary et al., 2015; Gunti and Notkins, 2015; Fernández-Quintero et al., 2019b). These probabilities are then shifted toward a smaller number of states which results in a reduction of possible binding partners.

Again, Figure 6C shows the reduced structural ensemble of the CDR-H3 loop upon affinity maturation. Figure 7 characterizes the plasticity of the naïve and matured 48G7 Fabs and reflects the substantial rigidification of the CDR-H3 loop in the matured Fab.

In all in detailed investigated antibody fragments we observe a significant decrease in flexibility and plasticity upon affinity maturation accompanied by strong population shifts toward the binding competent state. The free energy surfaces of the CDR-H3 loop do not only show a reduction in conformational space, but also reveal a smaller number CDR-H3 loop conformational states in solution. This is reduction in conformational diversity is reflected by narrower and deeper minima, while the naïve antibodies have broader and shallower free energy landscapes. Thus, germline antibodies–before maturation–are able to still adopt various distinct conformations, each of which is able to recognize different antigens.

## Conclusion

For 10 pairs of antibodies we observed a substantial rigidification in flexibility and plasticity upon affinity maturation, in particular for the CDR-H3 loop. Molecular plasticity plays a crucial role in all processes involving molecular recognition. In our manuscript we present for the first time a method to quantify and localize plasticity on an atomistic level. We show that this method is in excellent agreement with 3PEPS spectroscopy. Additionally, we employ this new method to affinity maturation of antibodies, showing for 10 pairs of antibodies, that affinity maturation goes hand in hand with a reduction of plasticity and flexibility. As our method allows for localization, we are even able to identify the areas of reduced plasticity. Consequently, we are able to show that for all 10 pairs of antibodies of different CDR-H3 loop lengths specificity is linked to rigidity. For all affinity maturation studies kinetics and thermodynamics were reconstructed and revealed for the naïve Fabs broader and shallower free energy surfaces, while the matured Fabs showed small and distinct minima. All studied affinity maturation Fabs follow the paradigm of conformational selection, because even without the antigen present the binding competent state is present in solution. In summary, we do not only demonstrate a generalizable method to characterize and localize molecular plasticity in detail, but we also strongly link it to a general principle in antibody-antigen recognition.

## Data Availability Statement

All datasets presented in this study are included in the article/Supplementary Material.

## Author Contributions

MF-Q, JL, LB, and KL conceived the study. MF-Q, JL, LB, and FW performed the research under the supervision of KL, and curated and analyzed the data. CS contributed to the study in visualizing and analyzing the data. MF-Q drafted the manuscript. All authors critically reviewed the manuscript.

## Conflict of Interest

The authors declare that the research was conducted in the absence of any commercial or financial relationships that could be construed as a potential conflict of interest.

## Abbreviations

CDR: complementary determining region
Fab: antigen binding fragment
Fv: antibody variable fragment
MD: molecular dynamics
PCCA: perron cluster cluster analysis
RMSD: root mean square deviation
tICA: time-lagged independent component analysis
V_H_: heavy chain
V_L_: light chain.
