# Supplementary material for: Local and Global Rigidification Upon Antibody Affinity Maturation
Source: Front Mol Biosci. 2020 Aug 7;7:182. doi: 10.3389/fmolb.2020.00182 (PMC7426445; doi:10.3389/fmolb.2020.00182)
Supplement: Supplementary file 1 [file Table_1.DOCX]

**Supporting Information**
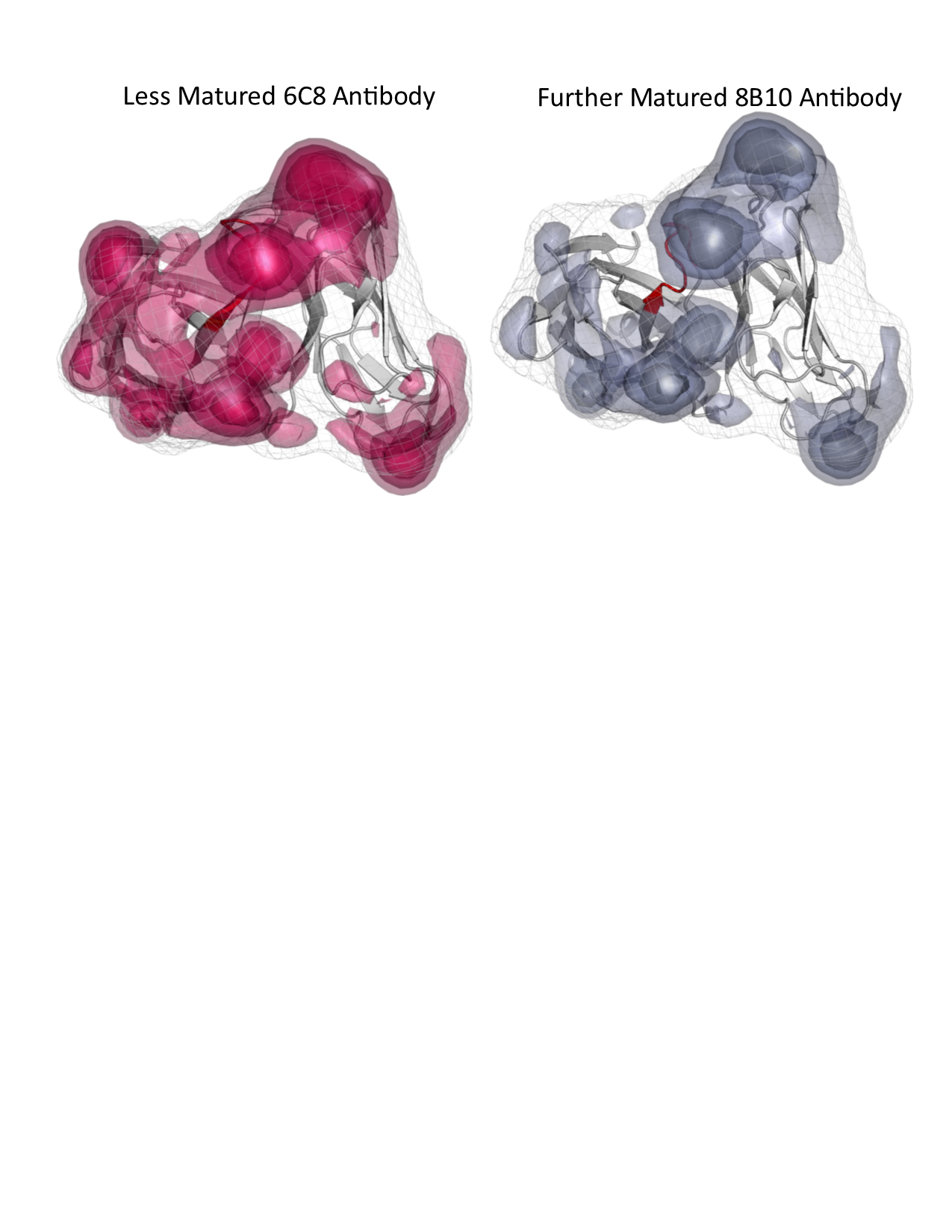


SI Figure S1: Visualized differences in surface plasticity of the less matured 6C8 and the further matured 8B10 antibody supported by experimental information.

Table 1: Mean values and confidence intervals for probabilities calculated from the Bayesian MSM. The confidence intervals are calculated at a confidence level of 95% of the D44.1 and F10.6.6 antibodies.

| **D44.1 Antibody** | mean | lower limit | upper limit |
| --- | --- | --- | --- |
| Macrostate | 0.38 | 0.35 | 0.41 |
| Macrostate | 0.34 | 0.31 | 0.37 |
| Macrostate | 0.25 | 0.22 | 0.28 |
| Macrostate | 0.03 | 0.02 | 0.04 |
| **F10.6.6 Antibody** | mean | lower limit | upper limit |
| Macrostate | 0.57 | 0.54 | 0.60 |
| Macrostate | 0.38 | 0.36 | 0.40 |
| Macrostate | 0.05 | 0.03 | 0.07 |

Table 2: Mean values and confidence intervals for probabilities calculated from the Bayesian MSM. The confidence intervals are calculated at a confidence level of 95% of the 28B4 antibodies.

| **28B4 Antibody (naive)** | mean | lower limit | upper limit |
| --- | --- | --- | --- |
| Macrostate | 0.82 | 0.79 | 0.85 |
| Macrostate | 0.11 | 0.09 | 0.14 |
| Macrostate | 0.07 | 0.05 | 0.09 |
| **28B4 Antibody (mature)** | mean | lower limit | upper limit |
| Macrostate | 0.86 | 0.81 | 0.90 |
| Macrostate | 0.14 | 0.11 | 0.17 |

Table 3: Mean values and confidence intervals for probabilities calculated from the Bayesian MSM. The confidence intervals are calculated at a confidence level of 95% of the 48G7 antibodies.

| **48G7 Antibody (naive)** | mean | lower limit | upper limit |
| --- | --- | --- | --- |
| Macrostate | 0.42 | 0.39 | 0.46 |
| Macrostate | 0.23 | 0.21 | 0.27 |
| Macrostate | 0.22 | 0.18 | 0.26 |
| Macrostate | 0.13 | 0.09 | 0.17 |
| **48G7 Antibody (mature)** | mean | lower limit | upper limit |
| Macrostate | 0.76 | 0.74 | 0.79 |
| Macrostate | 0.24 | 0.22 | 0.27 |


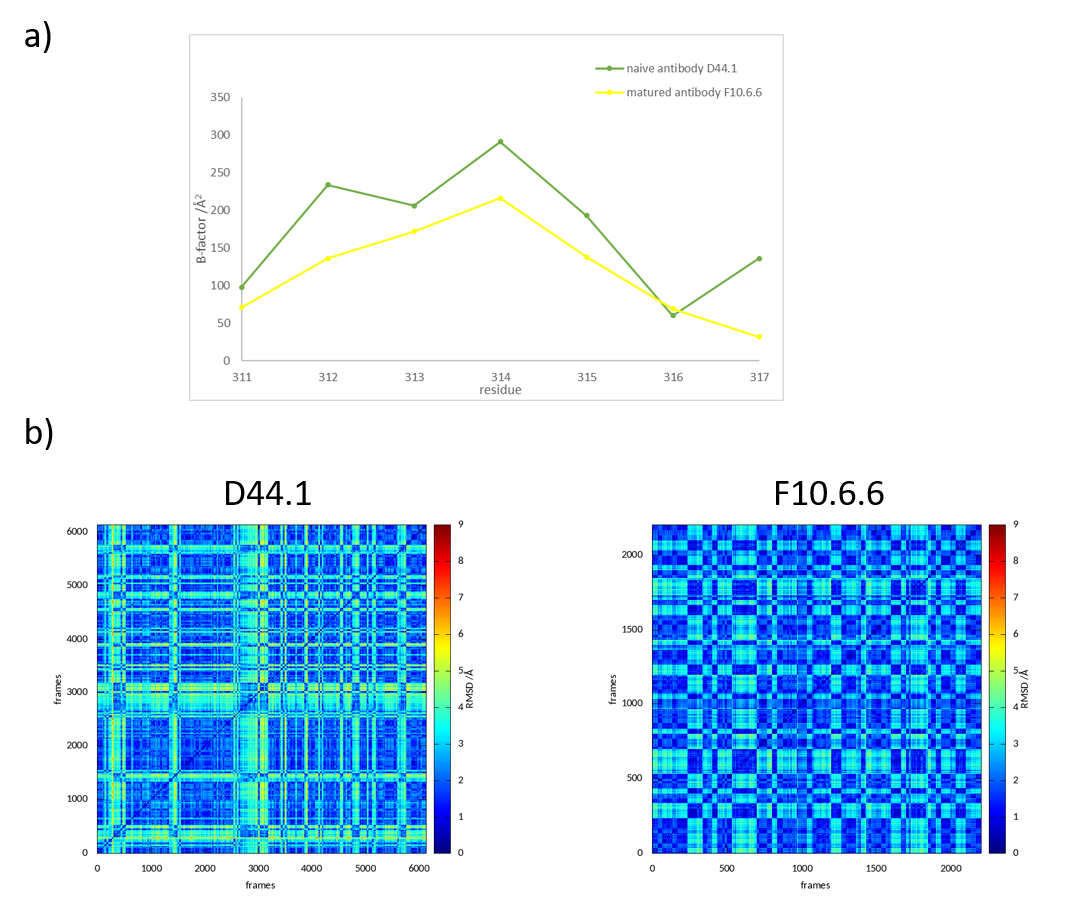


SI Figure S2: Cα B-factors (a) und 2D-RMSD plots (b) of the naive (D44.1 – left) and the matured (F10.6.6 – right) antibody CDR -H3 loop.


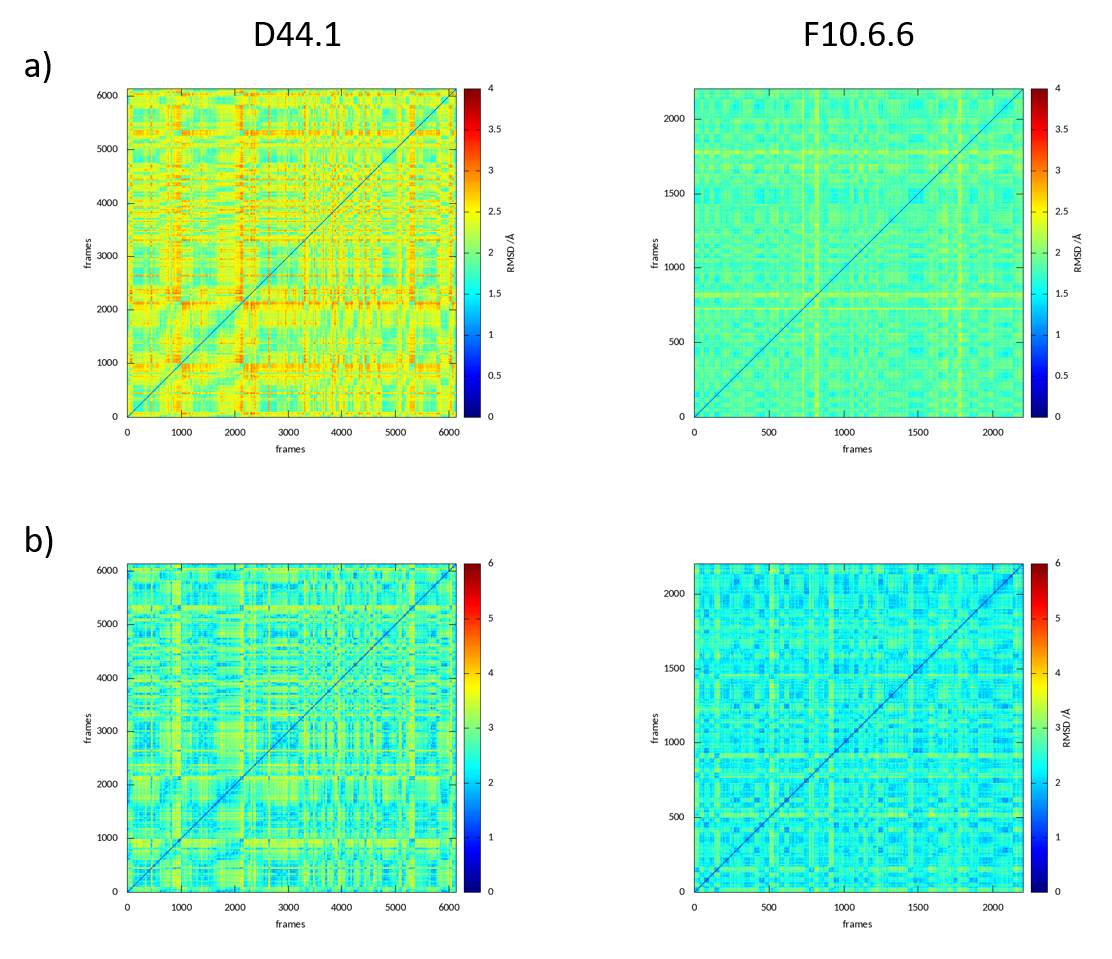


SI Figure S3: 2D-RMSD plots of the naive (D44.1 – left) and the matured (F10.6.6 – right) antibody. (a) 2D-RMSD plots of the paratope (all six CDR loops) and 2D-RMSD plots of the whole variable fragment (b).


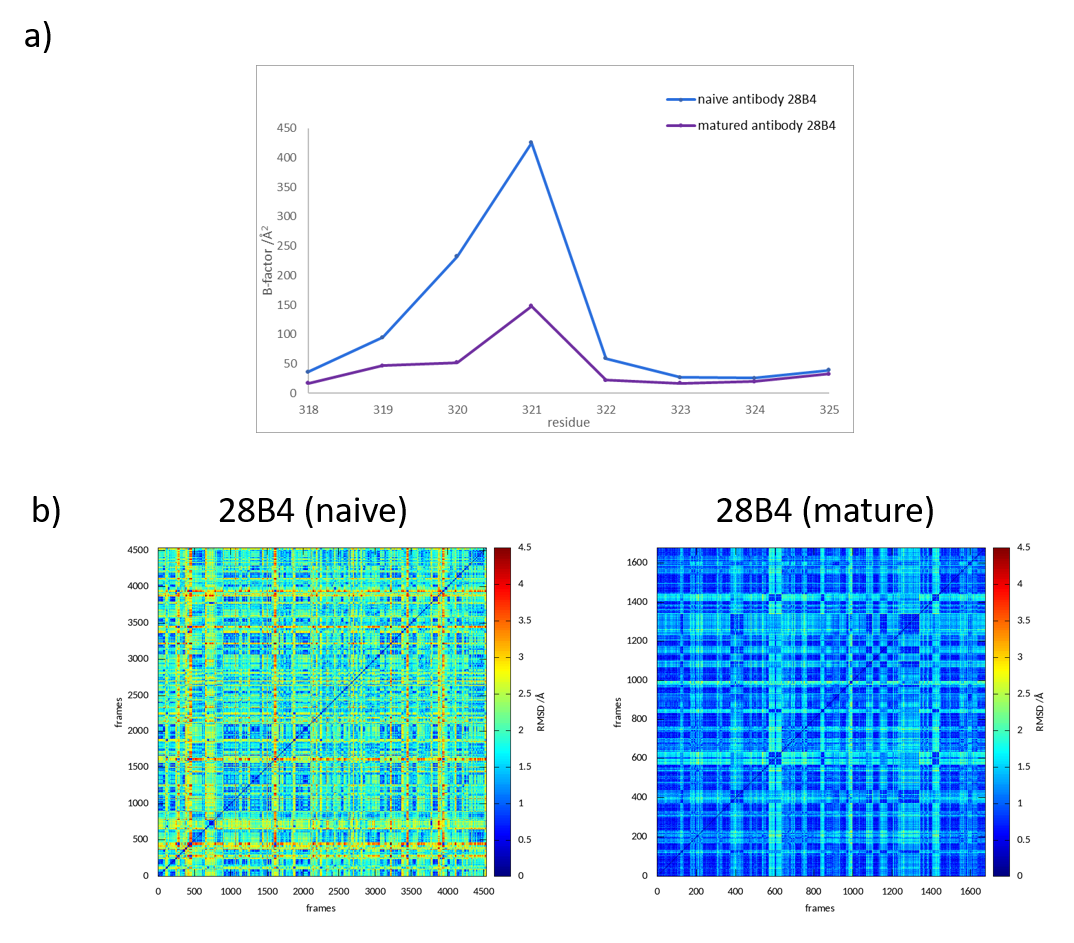


SI Figure S4: Cα B-factors (a) und 2D-RMSD plots (b) of the naive and the matured 28B4 antibody CDR -H3 loop.


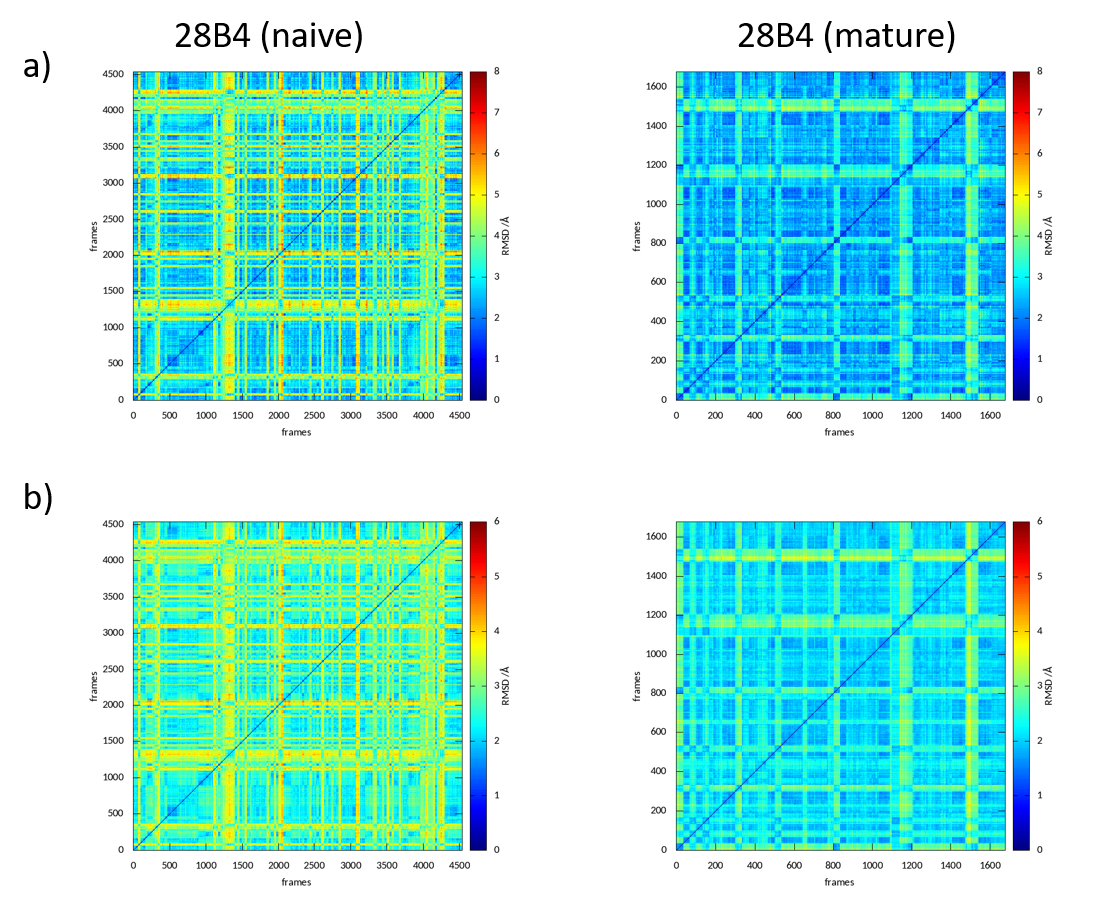


SI Figure S5: 2D-RMSD plots of the naive and the matured 28B4 antibody. (a) 2D-RMSD plots of the paratope (all six CDR loops) and 2D-RMSD plots of the whole variable fragment (b).


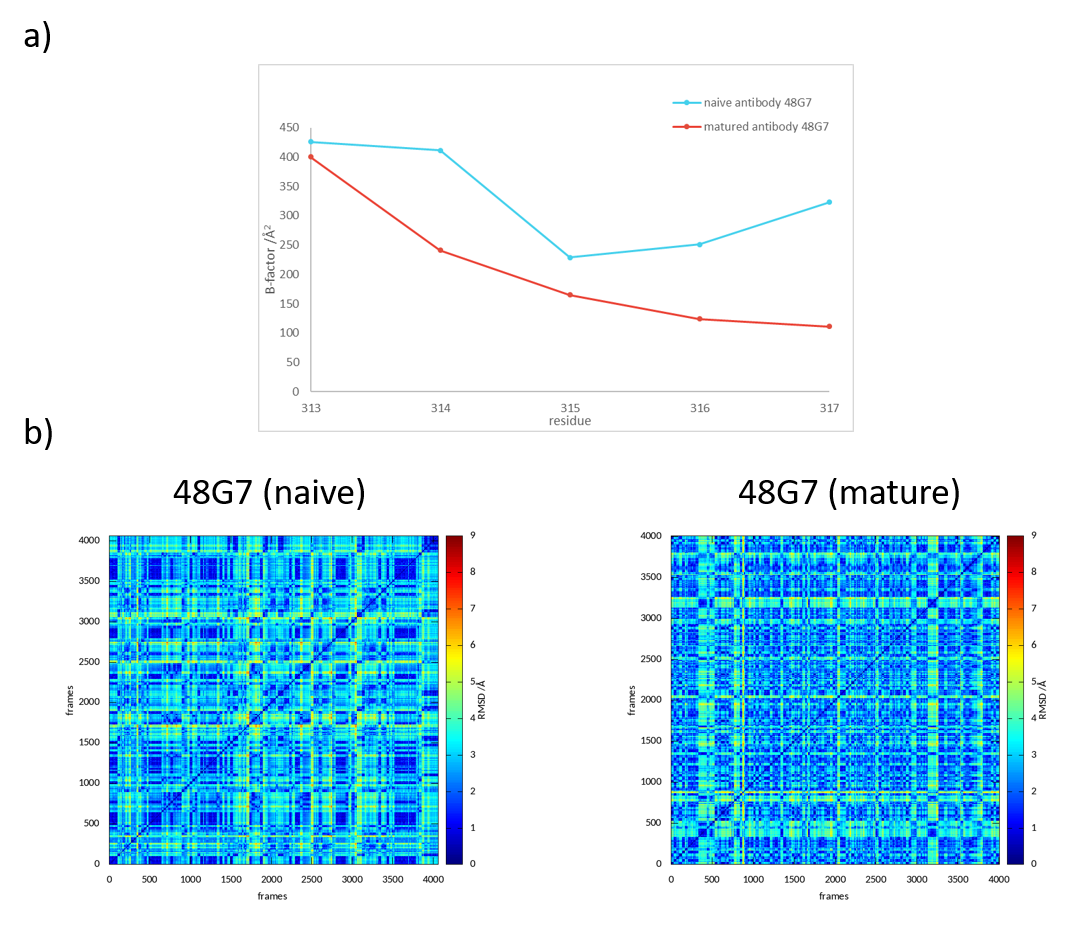


SI Figure S6: Cα B-factors (a) und 2D-RMSD plots (b) of the naive and the matured 48G7 antibody CDR -H3 loop.


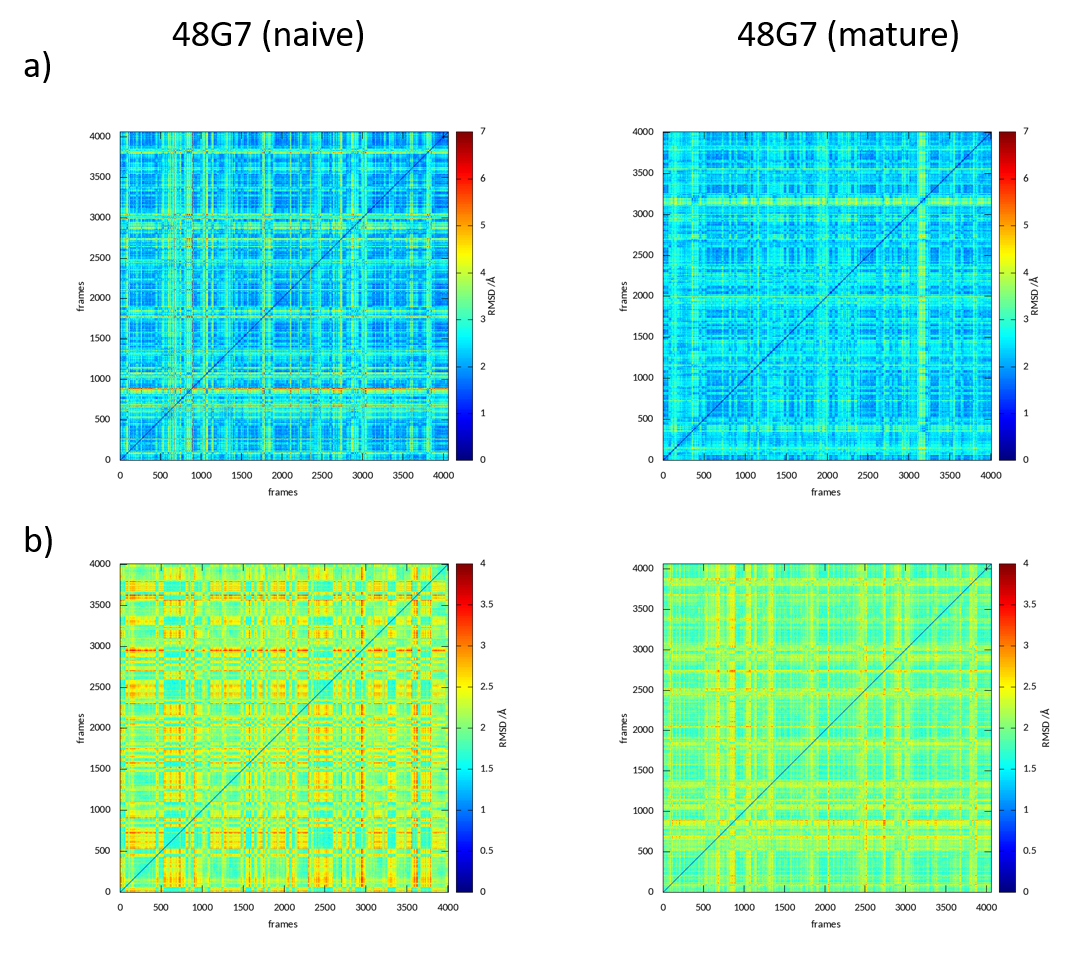


Figure 7: 2D-RMSD plots of the naive and the matured 48G7 antibody. (a) 2D-RMSD plots of the paratope (all six CDR loops) and 2D-RMSD plots of the whole variable fragment (b).

Summary and more detailed description of all investigated antibodies:

### Antibody 28B4

Antibody 28B4 catalyzes a periodate-dependent oxidation of sulfide to sulfoxide, whereby the hapten (1-[N-4’-nitrobenzyl-N-4’-carboxybutylamino] methylphosphonic acid) was generated to mimic the transition state of this reaction. The available crystal structures of the germline Fab fragment complexed with the hapten and without the hapten present as well as the affinity matured counterparts (PDB codes: 1FL6, 1FL5, 1KEL and 1KEM) were used as starting structures for metadynamics simulations. Nine mutations are introduced in the variable domain during affinity maturation, two in the light chain and seven in the heavy chain, two of them being far away from the binding region next to the constant domain. Three of these mutated residues of the mature antibody, Asn^35H^, Lys^56H^ and Trp^101H^ directly interact with the hapten. A list of all mutations can be seen in Table 4. A decrease of flexibility and changes in the binding geometry of the antigen due to these mutations lead to increased complementarity and therefore affinity between the antibody and the hapten. The mutations affect the affinity towards the antigen by direct contact as well as through secondary sphere interactions.

Table 4: Structures of naïve and affinity matured antibody 28B4. The mutations that were introduced in the course of affinity maturation are represented as sticks and colored according to the table on the left, the CDR region is colored in dark grey.

| Naïve | Affinity matured | Naïve | Affinity matured |
| --- | --- | --- | --- |
| Ser^L25^ | Phe^L25^ | 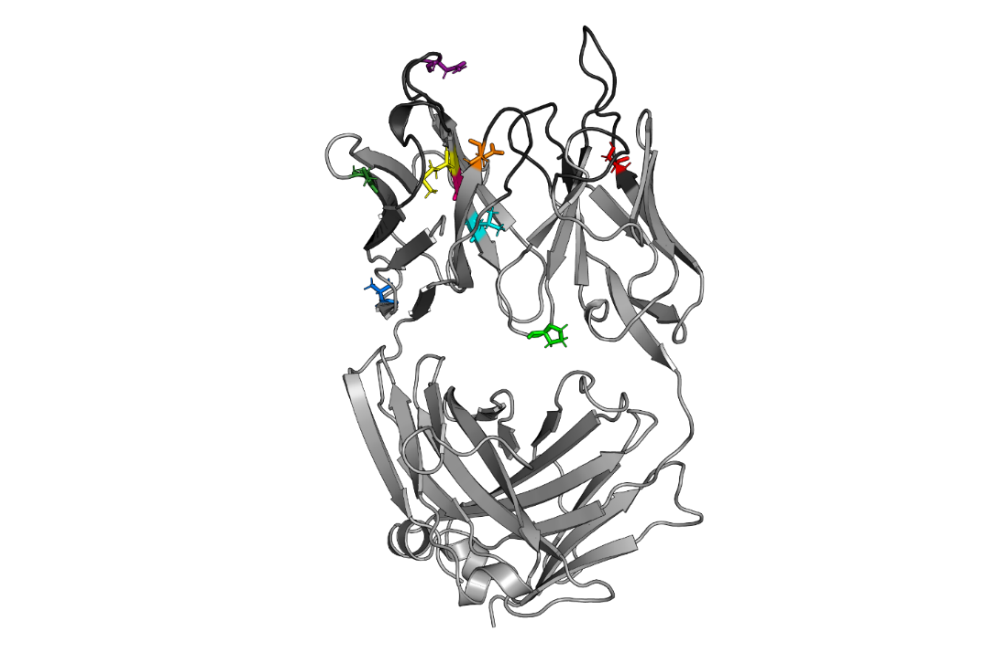 | 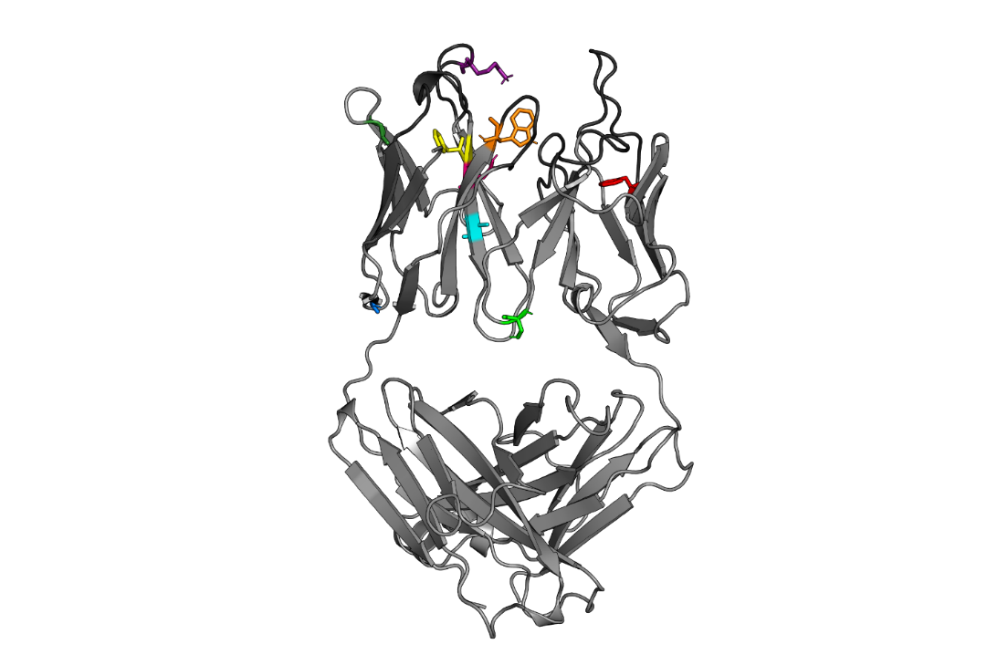 |
| Pro^L40^ | Ser^L45^ |  |  |
| Val^H12^ | Gly^H12^ |  |  |
| Met^H34^ | Phe^H34^ |  |  |
| Ser^H35^ | Asn^H35^ |  |  |
| Val^H37^ | Ala^H37^ |  |  |
| Asn^H53^ | Lys^H56^ |  |  |
| Ser^H76^ | Gly^H79^ |  |  |
| Asp^H95^ | Trp^H101^ |  |  |

### Antibody 48G7

The esterolytic antibody 48G7 catalyzes an ester and carbonate hydrolysis reaction, whereby the hapten 5-(para-nitrophenyl phosphonate)-pentanoic acid portrays the transition state. Available crystal structures that were used as starting structures for subsequent metadynamics simulations are the germline (i.e. not matured) Fab fragment in complex with the hapten and without the antigen present as well as the corresponding affinity matured structures. All crystal structures are deposited in the Protein Data Bank with the PDB codes 1AJ7, 2RCS, 1GAF and 1HKL, respectively. During the process of affinity maturation, nine mutations were introduced, three in the light chain and six in the heavy chain, depicted in Table 5. Although none of the mutated residues are in direct contact with the antigen, the affinity towards the hapten is raised by a factor of 30,000 due to fundamental changes in the structure induced by these mutations. Furthermore, the mutations induce a reorganization of hydrogen bonds, electrostatic and van der Waals interactions in the variable region of the antibody whereby side-chain and backbone flexibility may be diminished.

The differences between the crystal structures of bound and unbound matured antibody are very small (RMSD for Cα of the variable region is 0.39 Å), however for the germline antibody, binding of the hapten leads to larger changes in structure (RMSD for Cα of the variable region is 0.61 Å), especially residues in CDR-H3 are reorganized due to binding of the hapten. Thus it appears that the binding site gets pre-organized in the germline complex antibody and gets further optimized due to affinity maturation.

Furthermore, for antibody 48G7 each mutation was evaluated whether its enhanced affinity towards the hapten. It was found, that some mutations seem to be neutral on their own but contribute cooperatively to increasing affinity together with other mutations.

Table 5: Structures of naïve and affinity matured antibody 48G7. The mutations that were introduced in the course of affinity maturation are represented as sticks and colored according to the table on the left, the CDR region is colored in dark grey.

| Naïve | Affinity matured | Naïve | Affinity matured |
| --- | --- | --- | --- |
| Ser^L30^ | Asn^L30^ | 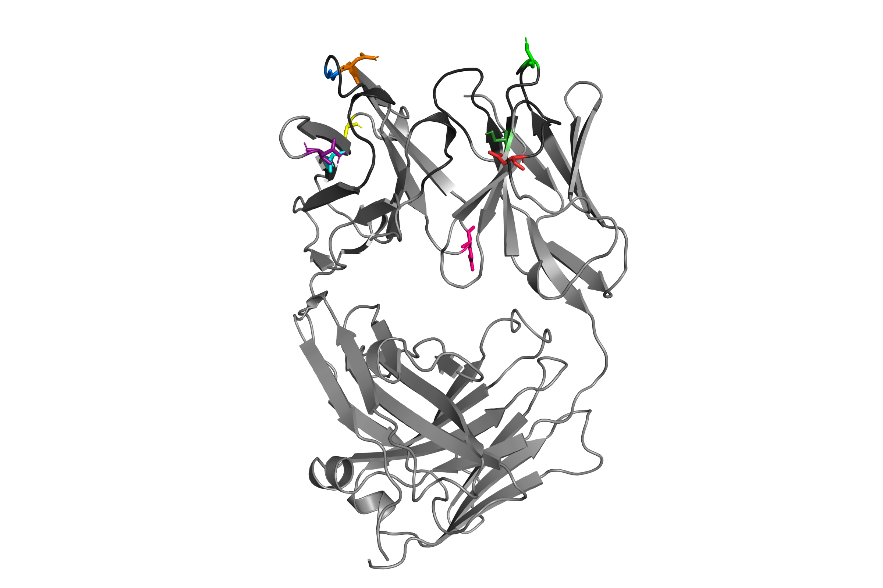 | 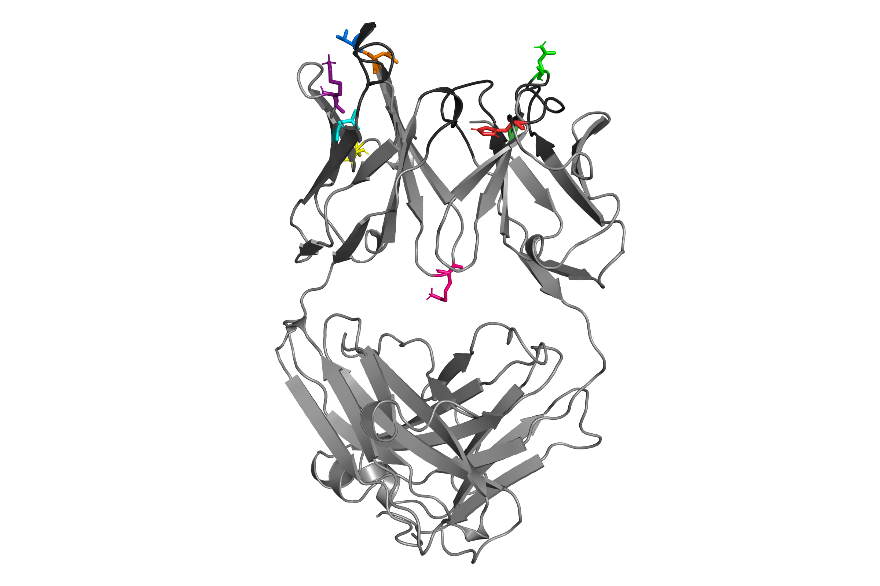 |
| Ser^L34^ | Gly^L34^ |  |  |
| Asp^L55^ | His^L55^ |  |  |
| Glu^H42^ | Lys^H42^ |  |  |
| Gly^H55^ | Val^H55^ |  |  |
| Asn^H56^ | Asp^H56^ |  |  |
| Gly^H65^ | Asp^H65^ |  |  |
| Asn^H76^ | Lys^H76^ |  |  |
| Ala^H78^ | Thr^H78^ |  |  |

### Antibodies D44.1 and F10.6.6

The two closely related antibodies D44.1 and F10.6.6 both bind the same lysozyme from hen egg white, with F10.6.6 being the more matured system having a 700-fold higher affinity towards lysozyme than D44.1. Both systems are murine monoclonal antibodies that are related in sequence and structure as they originate from the same germline gene rearrangement. However, they were produced with different immunization protocols. It seems that the two antibodies are at different stages or originate from different pathways of affinity maturation. The utilized crystal structures of the Fab fragments of antibodies D44.1 and F10.6.6 in complex and without the lysozyme present own the PDB-codes 1MLB, 1MLC, 2Q76 and 1P2C, respectively.

In sum, twenty mutations differ antibody D44.1 from antibody F10.6.6, seven of them being in the CDR loops. Details regarding these mutations can be found in Table 6. Due to the occurring mutations, structural changes yielded a stabilized V_H_-V_L_ interface with improvements in the binding situation towards the antigen and a tremendous raise in the affinity towards it. A significant increase in the number of non-covalent bonds between antibody and antigen as well as the bonds being closer and stronger was observed. The structural difference between bound and unbound form seems to be larger for the more matured antibody, resulting in a higher RMSD value.

It should be noted that the affinity maturation for anti-hapten antibodies is different from that of anti-protein antibodies, as the latter seems to be more complex due to two to three times more contacts between antibody and antigen.

Table 6: Structures of the naïve antibody D44.1 and the affinity matured antibody F10.6.6. The mutations that were introduced in the course of affinity maturation are represented as sticks and colored according to the table on the left, the CDR region is colored in dark grey.

| Naïve | Affinity Matured | Naïve | Affinity Matured |
| --- | --- | --- | --- |
| Val^L51^ | Thr^L51^ | 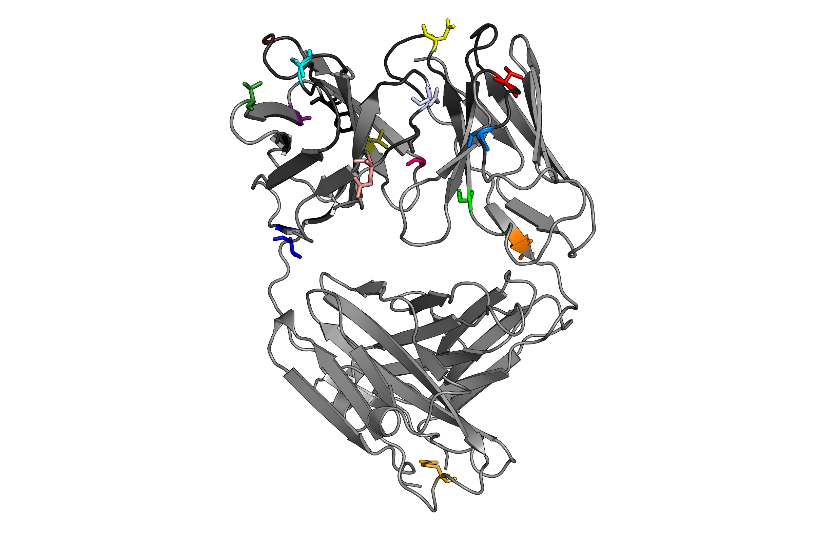 | 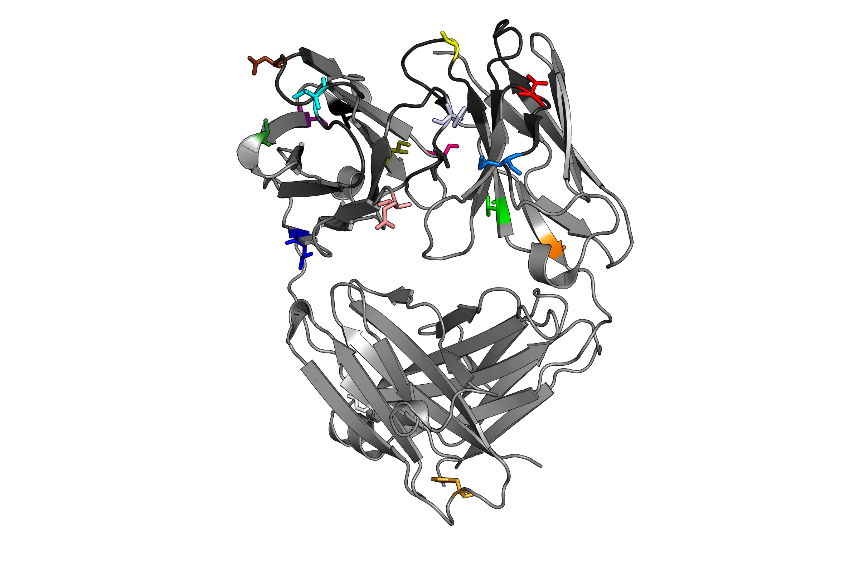 |
| Ser^L55^ | Met^L55^ |  |  |
| Met^L85^ | Val^L85^ |  |  |
| Asn^L92^ | Gly^L92^ |  |  |
| Glu^L105^ | Asp^L105^ |  |  |
| Gln^H1^ | Glu^H1^ |  |  |
| Val^H11^ | Leu^H11^ |  |  |
| Ser^H30^ | Thr^H30^ |  |  |
| Val^H37^ | Ile^H37^ |  |  |
| Gly^H44^ | Ser^H44^ |  |  |
| Gly^H56^ | Asp^H56^ |  |  |
| Phe^H64^ | Val^H64^ |  |  |
| Ala^H68^ | Val^H68^ |  |  |
| Thr^H74^ | Ala^H74^ |  |  |
| Asn^H102^ | Phe^H102^ |  |  |
| Gly^H104^ | Val^H104^ |  |  |
| Phe^H125^ | Tyr^H125^ |  |  |
| Arg^H191^ | Trp^H191^ |  |  |

### Antibody AZ28

Antibody AZ28 catalyzes an oxy-Cope rearrangement, a pericyclic reaction that rearranges hexadiene to aldehyde. The hapten (1S,2S,5S)2-(4-glutaridylbenzyl)-5-phenyl-1-cyclohexanol acts as a chair-like analogue of the transition state during this reaction. Crystal structures of the Fab fragments of germline and affinity matured antibody in complex as well as without the hapten present were used as starting points for metadynamics simulations (PDB codes: 1D6V, 1D5I, 1AXS and 1D5B). During the process of affinity maturation, six mutations were introduced, two in the light chain and four in the heavy chain, depicted in Table 7. Interestingly, for the mutation Ser^L34^ 🡪 Asn^L34^ it was shown that the affinity towards the hapten slightly increases but, on the other hand the catalytic efficiency decreases dramatically. Thus, it can be concluded that the germline antibody is a more effective catalyst than the affinity matured antibody mainly because of this specific mutation. The reaction is catalyzed with a 35-fold higher rate with the germline antibody although the affinity towards the antigen is 40-fold lower than in the matured one. The reason for the decreased catalytic activity of the matured antibody may be resulting from the hapten being bound in a catalytically unfavorable conformation. Due to the germline precursor showing higher conformational flexibility, the catalytic activity is higher but the binding affinity towards the hapten is decreased.

Table 7: Structures of naïve and affinity matured antibody AZ28. The mutations that were introduced in the course of affinity maturation are represented as sticks and colored according to the table on the left, the CDR region is colored in dark grey.

| Naïve | Affinity matured | Naive | Affinity matured |
| --- | --- | --- | --- |
| Ser^L34^ | Asn^L34^ | 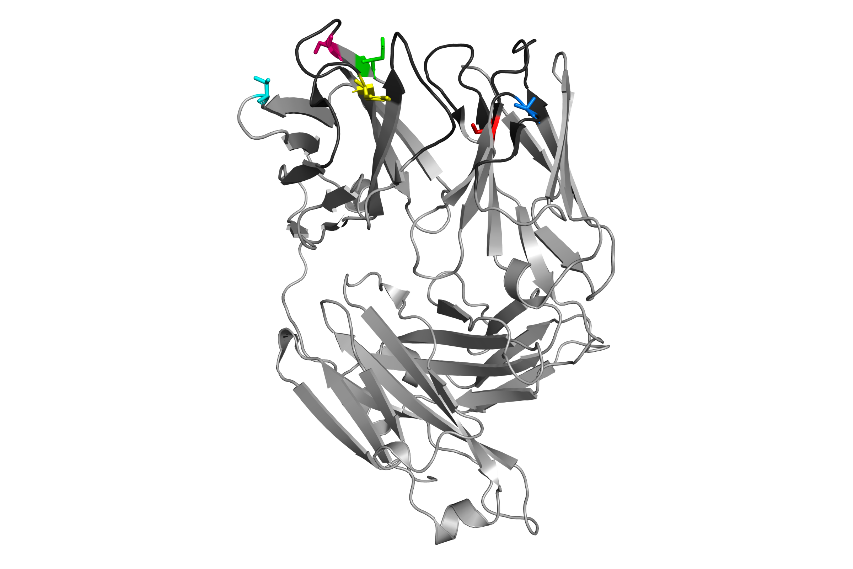 | 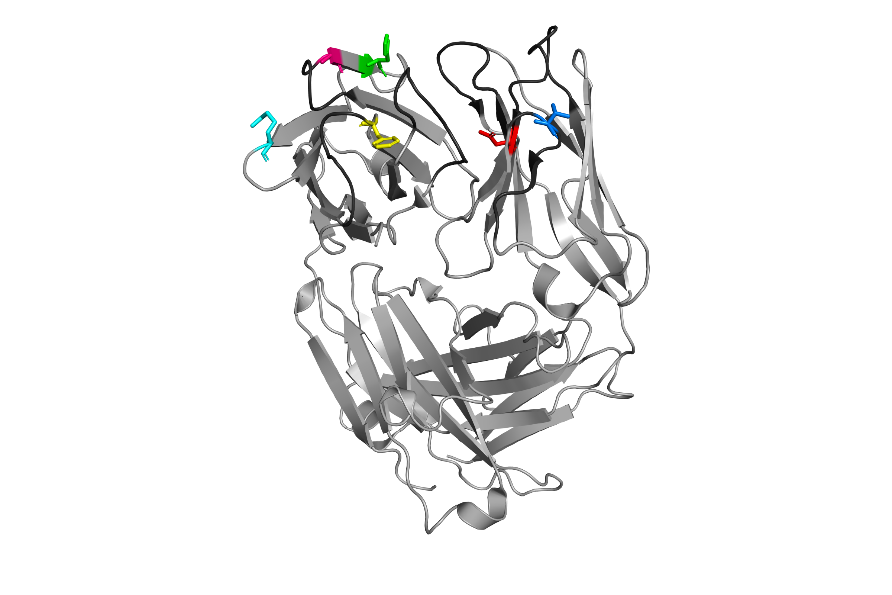 |
| Ala^L51^ | Thr^L51^ |  |  |
| Tyr^H32^ | Phe^H32^ |  |  |
| Ser^H56^ | Gly^H56^ |  |  |
| Asn^H58^ | His^H58^ |  |  |
| Thr^H73^ | Lys^H73^ |  |  |
|  | |  |  |

### Antibody 7G12

The ferrochelatase antibody 7G12 catalyzes the metalation of porphyrin, whereby mesoporphyrin, the hapten bound to the affinity matured antibody, portrays the configuration of the transition state. The crystal structures employed for metadynamics simulations include the germline Fab fragment complexed with the hapten N-methylmesoporphyrin as well as without the antigen present (PDB-codes 1N7M and 1NGZ) and the corresponding affinity matured antibodies in complex with mesoporphyrin as well as the counterpart without the hapten bound (PDB-codes 1NGW and 1NGY).

All in all, five mutations occurred during the affinity maturation process, two in the light chain and three in the heavy chain, listed in Table 8. Due to the maturation process, the affinity towards N-methylmesoporphyrin is 92-fold higher for the matured antibody which is represented in a higher catalytic activity as well. Particularly the mutation in CDR-H3 (Ser^H101^ 🡪 Met^H101^) leads to a conformational change in the backbone in the affinity matured antibody that allows binding the antigen in an optimized way.

The binding process seems to have a huge effect on the conformation of the CDR-H3 loop in the germline antibody, as this loop is massively involved in binding antigens. Due to the changes in the CDR-H3 loop, the binding site forms a cavity thereby clearly illustrating the flexibility of germline antibodies. On the contrary, the binding process in the affinity matured system does not cause large structural rearrangements, which can be interpreted as the structure being less flexible and pre-organized in the germline complex and further optimized for binding mesoporphyrin in the affinity maturation process. Thus, the flexibility of germline antibodies seems to be an important component for the immune system to develop antibodies that recognize a large variety of diverse antigens.

Table 8: Structures of naïve and affinity matured antibody 7G12. The mutations that were introduced in the course of affinity maturation are represented as sticks and colored according to the table on the left, the CDR region is colored in dark grey.

| Naïve | Affinity matured | Naïve | Affinity matured |
| --- | --- | --- | --- |
| Ser^L14^ | Thr^L14^ | 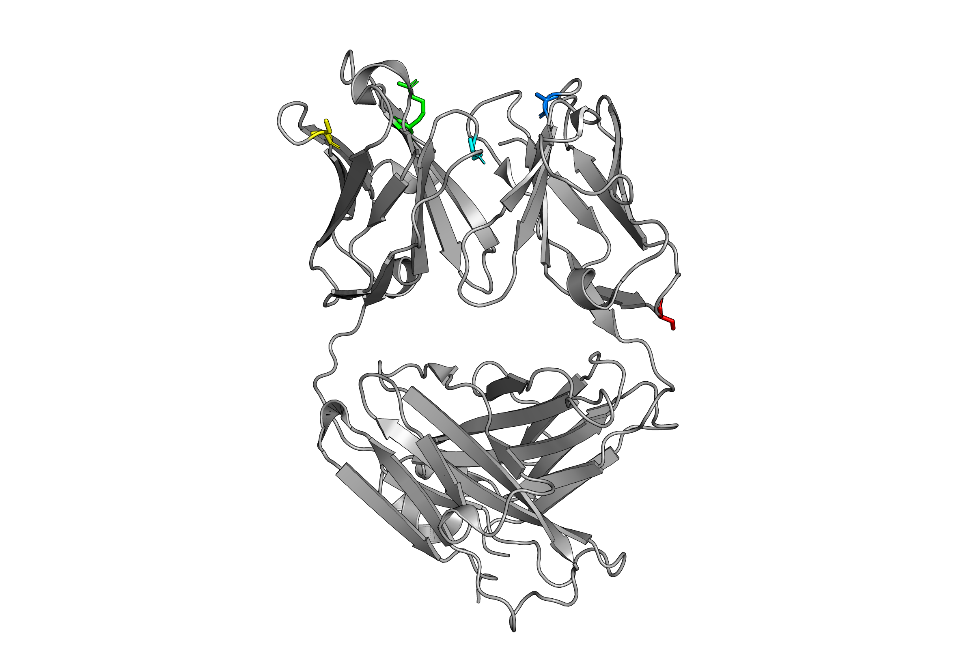 | 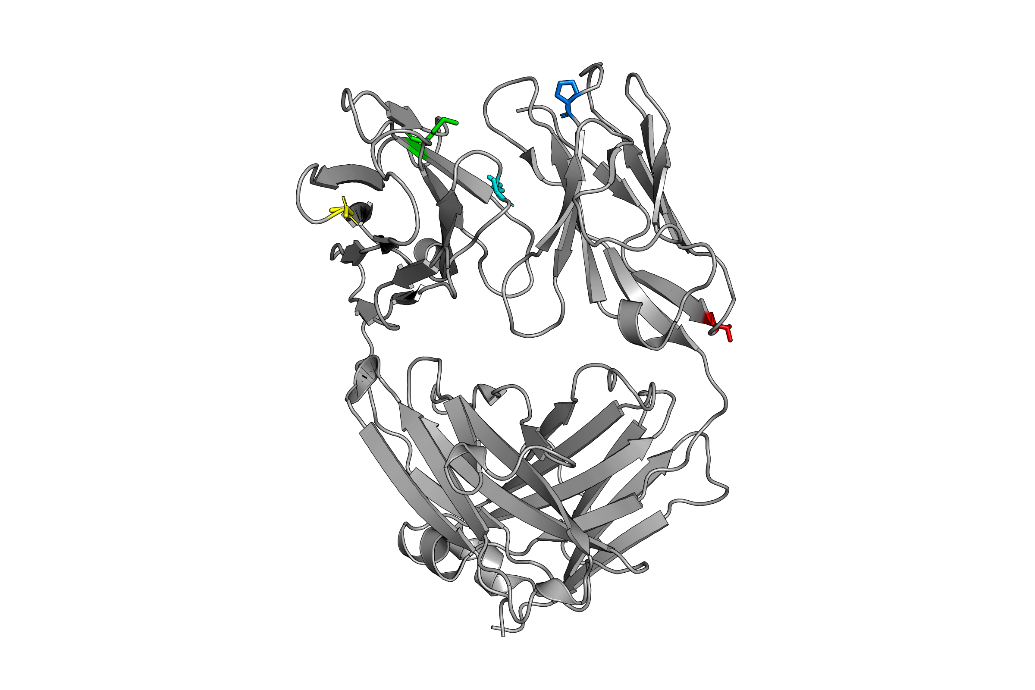 |
| Ala^L32^ | Pro^L32^ |  |  |
| Arg^H50^ | Met^H50^ |  |  |
| Ser^H77^ | Asn^H77^ |  |  |
| Ser^H101^ | Met^H101^ |  |  |
|  | |  |  |

### Antibodies H63 and H8

The two antibodies H63 and H8 bind the same anti-hen egg white lysozyme at the same site with H63 (PDB-code 1DQJ) being the less matured antibody as H8 (PDB-code 1NDG) has higher affinity towards the antigen. Both antibodies share the same V_L_ germline gene, therefore no mutation occurred in the light chain, however the heavy chain shows a lot of mutations, especially in the CDR region (a detailed table of all mutations can be seen in Table 9).

A wide range of residues in all CDR loops participate in contacting the lysozyme, whereby the hot spots being mainly responsible for the binding process are conserved. However, the affinity towards the antigen is 14-fold higher for the further matured antibody. It seems that the affinity is not increased due to electrostatic or van der Waals interactions as this is often the case in hapten-bound antibodies, but it is clearly observable that the CDR-H1 and CRD-H2 loops adopt different conformations during the process of affinity maturation, thereby enhancing the shape complementarity towards the antigen. The most important effect induced by the shift of the CDR loops is that the apolar surface is increased, especially due to the residues Tyr/Phe^H53^ and Tyr/Phe^H56^. This causes a tighter binding between antibody and antigen caused by a gain of hydrophobic interactions.

Another thing that should be noted is that haptens bind in a cleft between the CDR-H3 and CDR-L3 loop whereas proteins require the whole binding site, thereby interacting with all six CDR loops. Thus, the mechanisms, effects and consequences caused by affinity maturation are a more complex concept for protein-binding antibodies.

Table 9: Structures of the naïve antibody H63 and the affinity matured antibody H8. The mutations that were introduced in the course of affinity maturation are represented as sticks and colored according to the table on the left, the CDR region is colored in dark grey.

| Naïve | Affinity Matured | Naïve | Affinity Matured |
| --- | --- | --- | --- |
| Val^H29^ | Ile^H29^ | 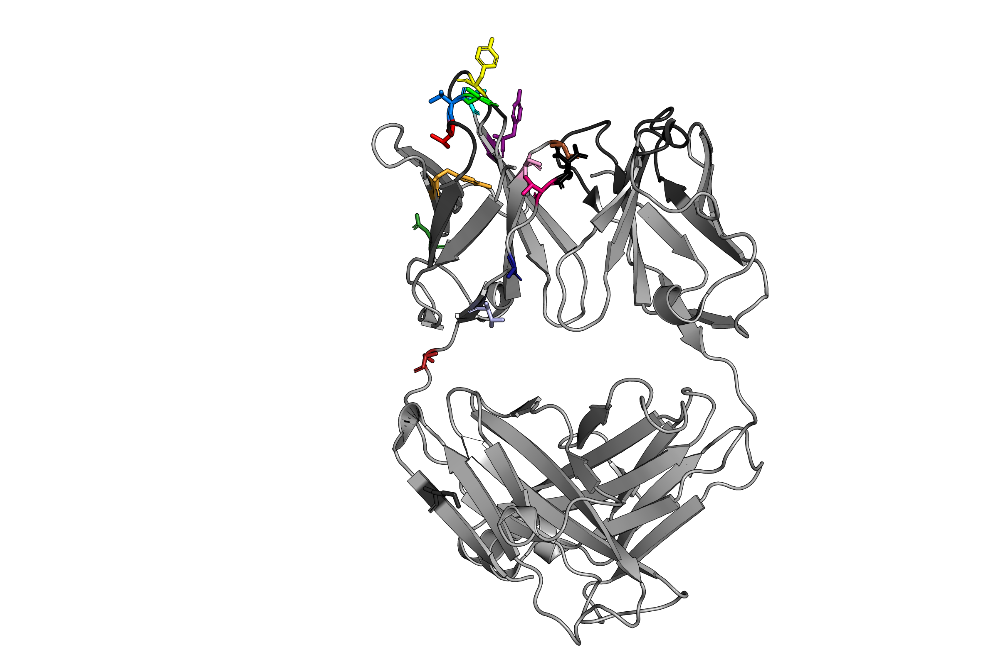 | 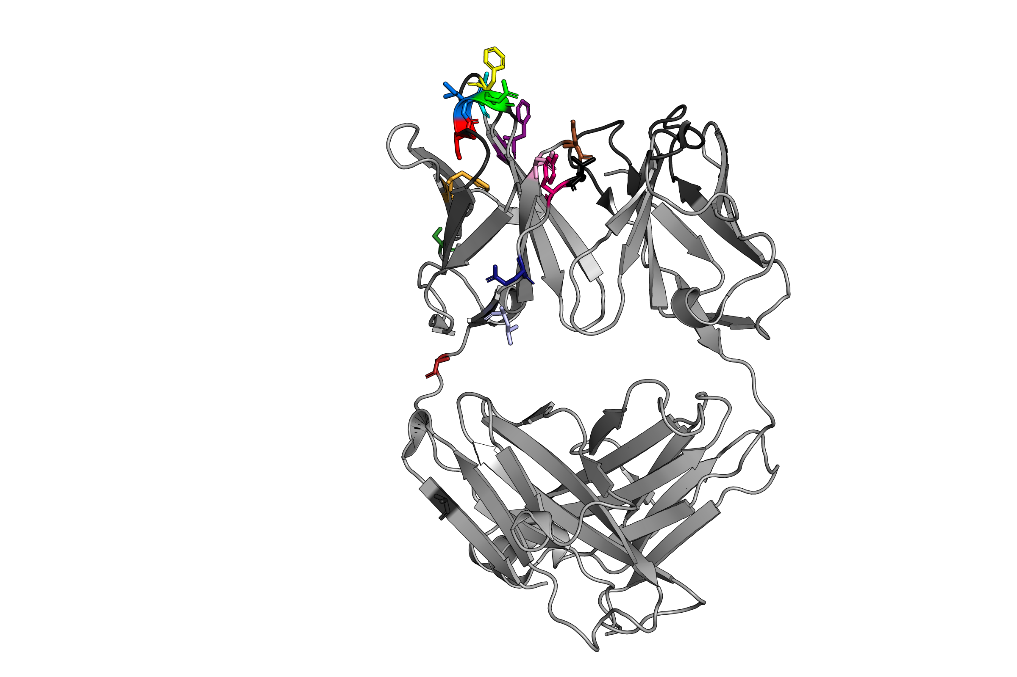 |
| Thr^H30^ | Ile^H30^ |  |  |
| Ser^H31^ | Arg^H31^ |  |  |
| Tyr^H53^ | Phe^H53^ |  |  |
| Ser^H56^ | Asn^H56^ |  |  |
| Tyr^H58^ | Phe^H58^ |  |  |
| Tyr^H78^ | His^H78^ |  |  |
| Asn^H83^ | Ser^H83^ |  |  |
| Ser^H97^ | Asn^H97^ |  |  |
| Gly^H99^ | Asp^H99^ |  |  |
| Asp^H101^ | Thr^H101^ |  |  |
| Val^H102^ | Tyr^H102^ |  |  |
| Ala^H105^ | Glu^H105^ |  |  |
| Thr^H108^ | Leu^H108^ |  |  |
| Ser^H113^ | Ala^H113^ |  |  |
| Lys^H216^ | Ala^H216^ |  |  |

### Antibodies S25-2 and S45-18

For carbohydrate-specific antibodies, the effect of class switching is usually not observed and affinity maturation does not take place either. Although carbohydrate-specific antibodies are important tools for immune surveillance, their affinity towards oligosaccharides is low.

Monoclonal antibody S25-2 shows affinity for the chlamydial family-specific terminal α(2🡪8)-linked 3-deoxy-D-manno-oct-2-ulosonic acid (Kdo) residues. The crystal structures used for metadynamics simulations include the Fab fragment complexed with the trisaccharide αKdo(2🡪8)-αKdo(2🡪4) as well as the Fab fragment without the antigen present (PDB-codes 1Q9Q and 1Q9K, respectively). Additionally, S25-2 is able to bind two other saccharides as well. The monoclonal antibody S45-18 on the other hand only binds the trisaccharide αKdo(2🡪4)-αKdo(2🡪4). The Fab fragments used for this antibody include the structure in complex (with the pentasaccharide αKdo(2🡪4)-αKdo(2🡪4)-αKdo(2🡪6)-β-GlcN-4P(1🡪6)-α-GlcN-1P that contains the α(2🡪4)-α(2🡪4) Kdo trisaccharide epitope) as well as without the antigen present (PDB-codes 1Q9W and 1Q9O, respectively). However, the two glucosamine phosphate molecules do not bind or interact with the antibody.

In sum, the two antibodies differ by five mutations in the light chain and several more mutations in the heavy chain (see Table 10 for a detailed description), including a completely modified CDR-H3 loop, differing in length as well as in the amino acids. Both antibodies originate from the same germline genes with antibody S45-18 being the further matured one. However, the regions of the CDR-H3 loop are based on different germline genes which explains the huge differences in this segment of the sequence. Finally, all the occurring mutations lead from the low-specificity antibody S25-2 to the highly specific antibody S45-18.

It was observed that binding of carbohydrate antigens is usually defined by a large amount of stacking interactions but in this case, mainly hydrogen bonds were found to be responsible for binding and only one stacking interaction in the S45-18 structure (Phe^H97^) was identified. Furthermore, it was found that the same residues in antibody S25-2 are in charge of binding different carbohydrate epitopes and, obviously, the modified CDR-H3 loop is the main factor for the high specificity of antibody S45-18 in contrast to S25-2 as the antigens contact the antibodies at residues in the CDR-L3, CDR-H1, CDR-H2 and CDR-H3 loops. Remarkably, no mutations occurred in CDR-L3 and H1.

Table 10: Structures of the naïve antibody S25-2 and the affinity matured antibody S45-8. The mutations that were introduced in the course of affinity maturation are represented as sticks and colored according to the table on the left, the CDR region is colored in dark grey.

| Naïve | Affinity Matured | Naive | Further matured |
| --- | --- | --- | --- |
| Ser^L7^ | Phe^L7^ | 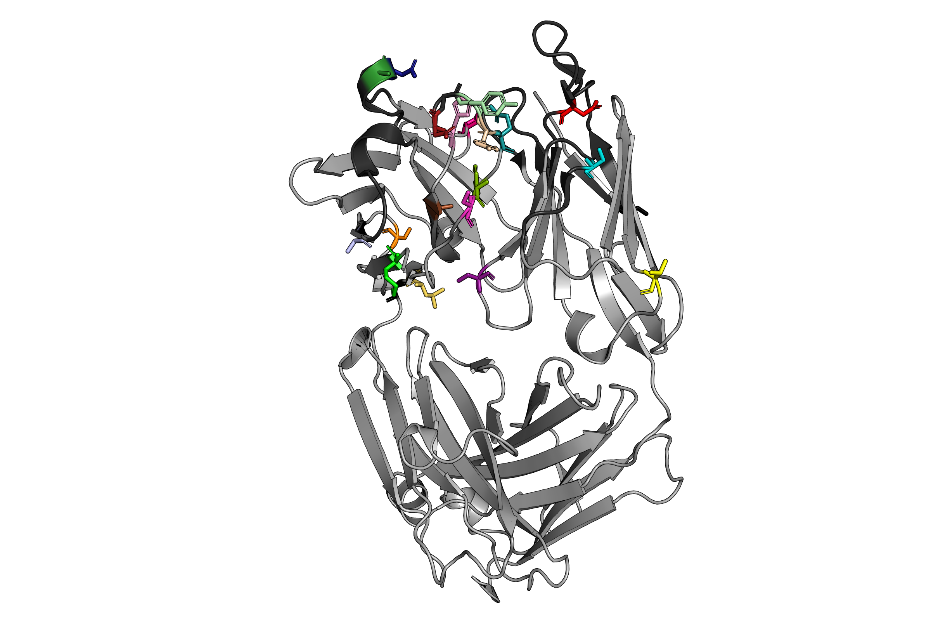 | 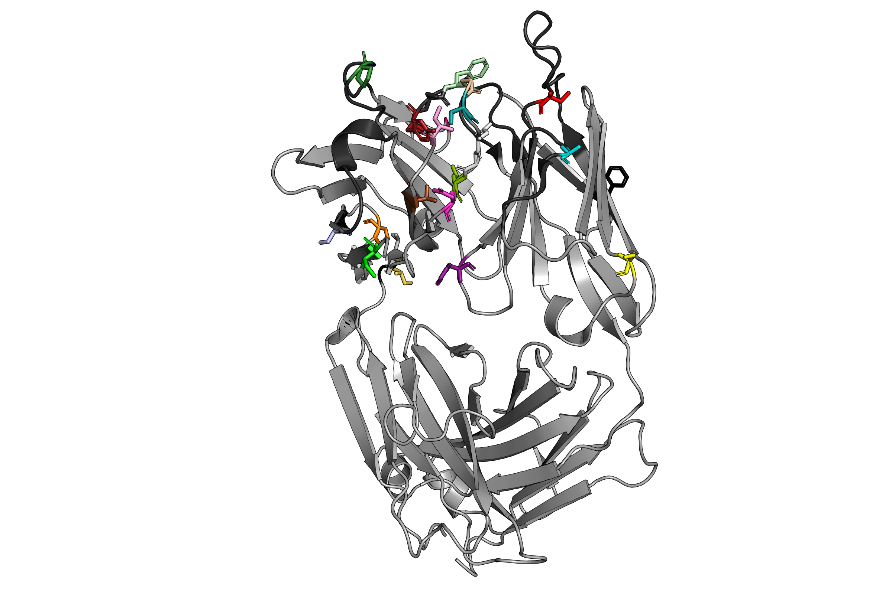 |
| Asn^L31^ | Ser^L31^ |  |  |
| Ser^L43^ | Phe^L43^ |  |  |
| Ser^L52^ | Ala^L52^ |  |  |
| Thr^L76^ | Ser^L76^ |  |  |
| Lys^H3^ | Ile^H3^ |  |  |
| Ser^H14^ | Pro^H14^ |  |  |
| Ala^H23^ | Ser^H23^ |  |  |
| Ala^H52C^ | Pro^H52C^ |  |  |
| Asn^H52D^ | Lys^H52D^ |  |  |
| Pro^H59^ | Ala^H59^ |  |  |
| Ala^H93^ | Val^H93^ |  |  |
| His^H96^ | Ile^H96^ |  |  |
| Asp^H97^ | Tyr^H96A^ |  |  |
| Gly^H98^ | Ser^H96B^ |  |  |
| Tyr^H99^ | Phe^H97^ |  |  |
| Tyr^H100^ | Gly^H98^ |  |  |
| Glu^H100A^ | Ser^H99^ |  |  |
| - | Asp^100A^ |  |  |
| - | Gly^H100B^ |  |  |
| Phe^H100C^ | Met^H100C^ |  |  |
| Ser^H100D^ | Asp^H100D^ |  |  |
| Leu^H106^ | Ser^H106^ |  |  |
| **Ala^H111^** | **Ser^H111^** |  |  |

### Antibodies 9-40 and 4-4-20

The crystal structures of antibodies 9-40 (PDB-code 1T66) and 4-4-20 (PDB-code 4FAB) that were used for metadynamics simulations are both Fab fragments complexed with fluorescein. While both of them bind the antigen in a similar manner, the affinity towards it differs strongly in the two systems. The interactions with the hapten are carried out by several aromatic amino acids, namely tryptophan and tyrosine but antibody 9-40 has a substantially lower affinity towards fluorescein compared to antibody 4-4-20. The reason for this observation lies in a more open binding site with lower complementarity that leads to weaker binding of the antigen, to be more precise in a 1000-fold reduction of affinity of antibody 9-40 compared to 4-4-20. The two antibodies were produced by distinct B cell lines but are of the same idiotypic family and differ by twelve somatic mutations in the variable domain, three of them occurred in the light chain, nine in the heavy chain. Due to these mutations a change in the structural conformation of antibody 4-4-20 led to the increased affinity towards fluorescein. The hugest differences were observed in the configuration of the CDR-H3 loop were five amino acids were mutated. Together with another two tyrosine residues, the mutation of Gly^H102^ to Tyr^H102^ keeps solvent molecules out of the antigen binding site in antibody 4-4-20. Another important factor for the enhanced affinity seems to be the mutation of His^L39^ to Arg^L39^ in CDR-L1 that directly interacts with the antigen. A list of all mutations can be found in Table 11.

Table 11: Structures of the naïve antibody 9-40 and the affinity matured antibody 4-4-20. The mutations that were introduced in the course of affinity maturation are represented as sticks and colored according to the table on the left, the CDR region is colored in dark grey.

| Naïve | Affinity Matured | Naïve | Affinity Matured |
| --- | --- | --- | --- |
| Asn^L33^ | Gln^L33^ | 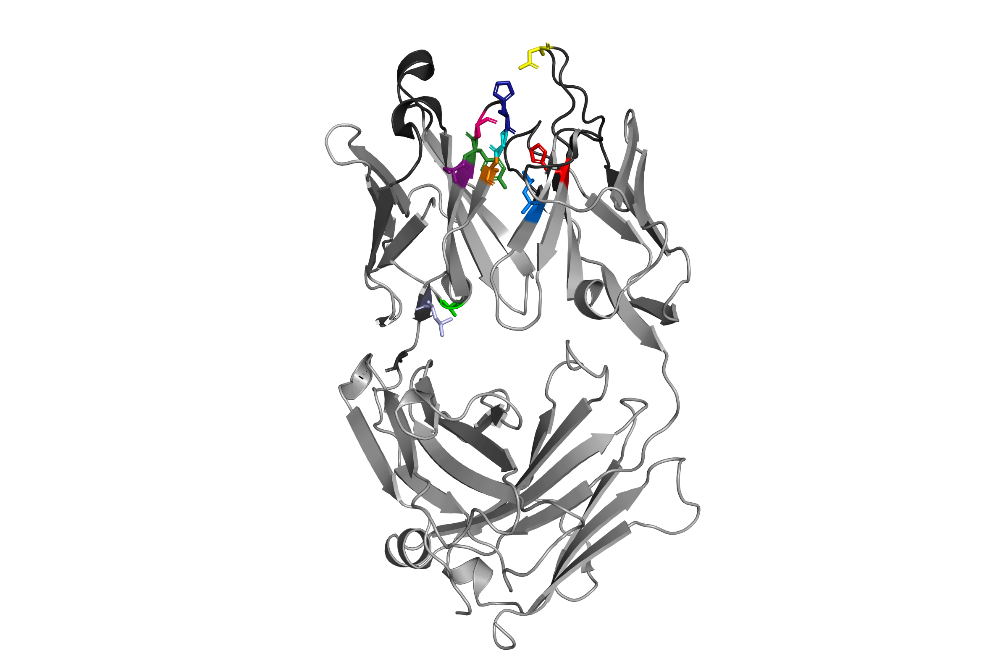 | 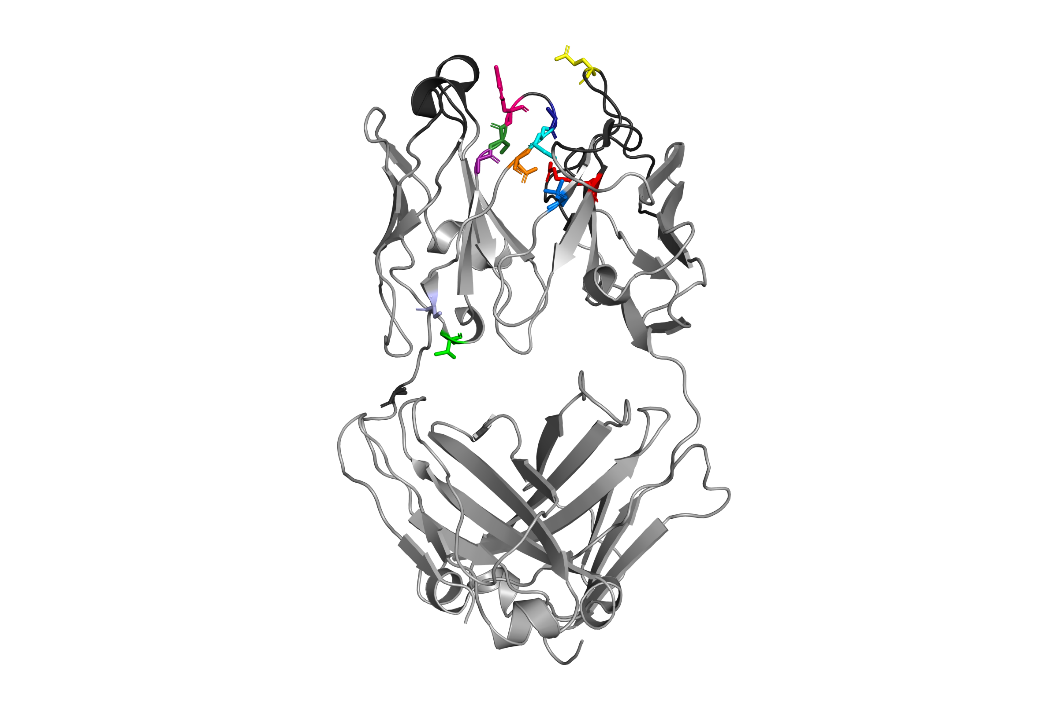 |
| His^L39^ | Arg^L39^ |  |  |
| Leu^L51^ | Val^L51^ |  |  |
| Ala^H90^ | Val^H90^ |  |  |
| Ser^H100^ | Gly^H100^ |  |  |
| Tyr^H101^ | Ser^H101^ |  |  |
| Gly^H102^ | Tyr^H102^ |  |  |
| His^H104^ | Gly^H104^ |  |  |
| Gly^H105^ | Met^H105^ |  |  |
| Ala^H106^ | Asp^H106^ |  |  |
| Leu^H113^ | Ser^H113^ |  |  |
| Ala^H118^ | Ser^H118^ |  |  |

### Antibodies 6C8 and 8B10

The crystal structures of antibodies 6C8 (PDB-code 4NJA) and 8B10 (PDB-code 4NJ9) are Fab fragments in complex with the chromophoric antigen 8-methoxypyrene-1,3,6-trisulfonate (MPTS). Both antibodies have diverged from a common ancestral germline and differ from each other by six mutations in the variable domains, two in the light chain and three in the heavy chain (a detailed table of all mutations can be found inTable 12). Antibody 8B10 binds the antigen with much higher affinity (K_D_ ~ 100 nm) compared to antibody 6C8 (K_D_ ~ 600 nm) which was also reflected in an ELISA experiment as antibody 6C8 binds several other proteins with similar or even higher affinity as MPTS whereas antibody 8B10 barely recognizes any other proteins and therefore seems to be more specific for MPTS.

The differences between the crystal structures of the antibodies in complex with the antigen are very small (RMSD for Cα of the variable domains is 0.42 Å) and MPTS is bound in a shallow pocket in both cases with residues of CDR-H3, CDR-L1 and CDR-L2 being in contact with the antigen. Five hydrogen bonds emerge with two of the three sulfonate groups of MPTS in both antibody complexes that seem to be important for their specificity. The mutation Asn^L30^ 🡪 Ile^L30^ in CDR-L1 results in an additional hydrogen bond with the third sulfonate group of MPTS with antibody 6C8. The biggest structural differences are within CDR-H1 and CDR-H2, which do not directly form part of the MPTS binding interface.

While the complex of antibody 6C8 with MPTS is very rigid and exhibits only low plasticity, 6C8 is nevertheless polyspecific suggesting the free antibody to be more flexible than its more mature counterpart 8B10 that shows higher affinity and specificity towards the antigen and no plasticity. Due to the mutations in antibody 8B10, the structure is highly anelastic as the mutated residues form several stabilizing hydrogen bonds that are not present in the less matured antibody.

Table 12: Structures of the less matured antibody 6C8 and the further matured antibody 8B10. The mutations that were introduced in the course of affinity maturation are represented as sticks and colored according to the table on the left, the CDR region is colored in dark grey.

| Naïve | Affinity Matured | Naïve | Affinity Matured |
| --- | --- | --- | --- |
| Asn^L30^ | Ile^L30^ | 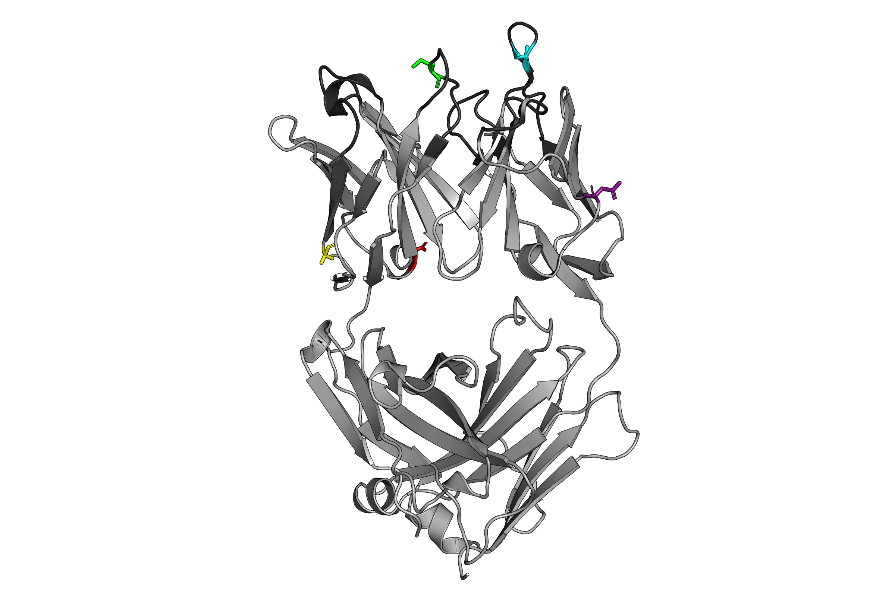 | 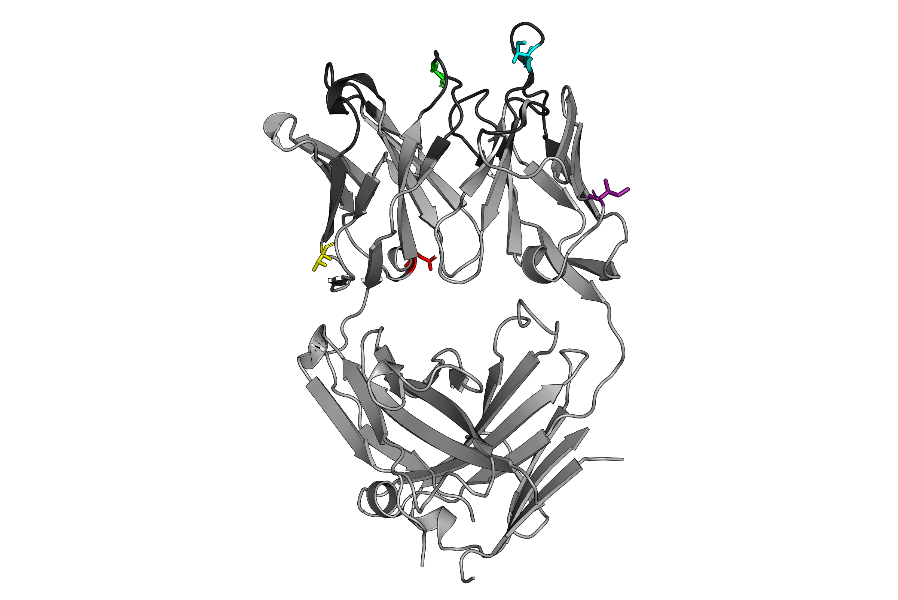 |
| Asn^L76^ | Ile^L76^ |  |  |
| Ala^H16^ | Thr^H16^ |  |  |
| Asp^H85^ | Glu^H85^ |  |  |
| Ile^H100^ | Ser^H100^ |  |  |
|  | |  |  |

### aDabi-Fab antibodies

Idarucizumab, also referred to as aDabi-Fab1 is a humanized Fab-fragment that is used as anti-anticoagulant as it binds the thrombin inhibitor dabigatran.^50^ When searching for backup clinical candidates to aDabi-Fab1, several mutants were identified. One of these mutants, aDabi-Fab2, was crystallized without the antigen as well as in complex with dabigatran and deposited in the Protein Data Bank with the PDB-codes 4YGV and 4YHI, respectively. The overall structures of the bound and the unbound antibody do not show any major differences. aDabi-Fab2 binds dabigatran in a different way compared to aDabi-Fab1, regarding the conformation as well as the involved interactions, as all CDR loops except L2 participate in binding the hapten with hydrophobic interactions, hydrogen bonds and a salt bridge.

As aDabi-Fab2 has a 90-fold weaker affinity towards the antigen compared to aDabi-Fab1, another mutant, aDabi-Fab3, was developed and crystallized in complex with the hapten dabigatran as well as without it (PDB-codes 4YHO and 4YHN), that provides higher binding affinity and improved capacities for inhibiting dabigatran. To enhance the affinity towards the hapten, additional interactions as well as an optimized shape complementarity are pivotal.

aDabi-Fab2 and aDabi-Fab3 differ by one mutation in the CDR-H3 loop, namely Tyr^H103^ 🡪 Trp^H103^, that results in improved shape complementarity due to a larger side chain being involved in the hydrophobic interaction with dabigatran. This single mutation led to a 10-fold higher binding affinity of aDabi-Fab3 towards dabigatran compared to aDabi-Fab2. The crystal structures of aDabi-Fab2 show no major differences compared to the aDabi-Fab2 structures. Even the hydrogen bond of the mutated amino acid remains unchanged, however, due to tryptophan having a larger side chain, the interaction is tighter.


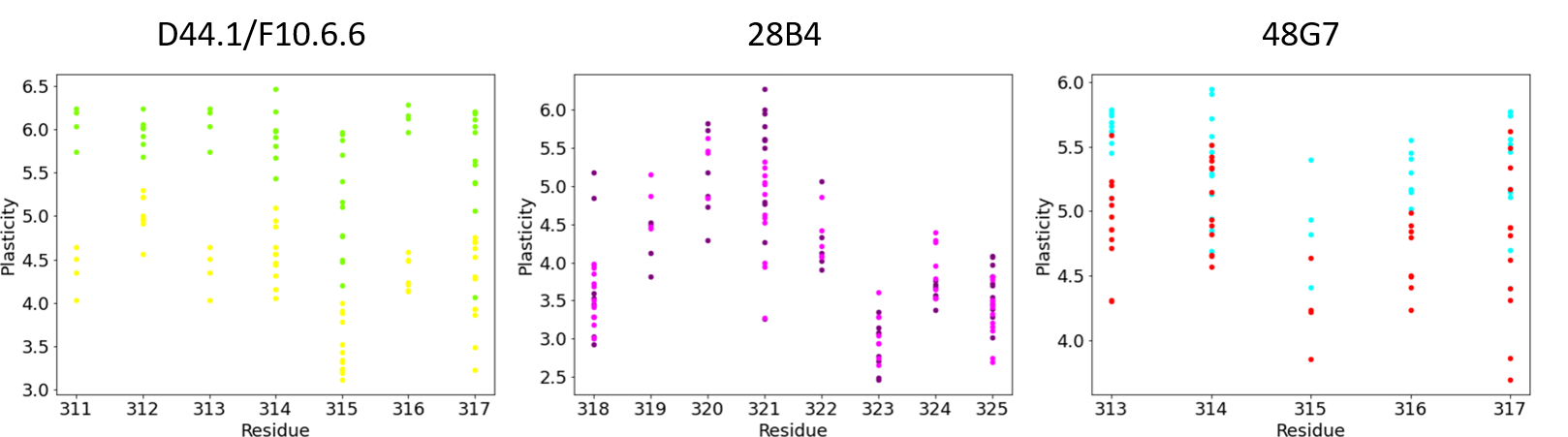


Figure 8: Localized CDR-H3 loop surface plasticity upon affinity maturation. The dots are color-coded according to the figures in the main manuscript. These plots clearly depict a substantial rigidification of plasticity upon affinity maturation for the CDR-H3 loops.

Table 13: Overall changes in surface plasticity upon affinity maturation. The color-coding corresponds to the main figures in the manuscript. The rigidification of the variable fragment can be clearly seen in the last column describing the percentage of rigidification, which is calculated from the individual plasticity.

| **Antigen** | **Naïve**  **(PDB Codes)** | **Plasticity** | **Matured**  **(PDB Codes)** | **Plasticity** | **Percentage of Rigidification** |
| --- | --- | --- | --- | --- | --- |
| D44.1/F10.6.6 | 1MLB | 5.49 ± 0.96 | 1P2C | 3.57 ± 0.72 | 35% |
| 28B4 | 1FL6 | 4.15 ± 0.83 | 1KEL | 2.62 ± 0.96 | 37% |
| 48G7 | 1AJ7 | 6.25 ± 0.94 | 1GAF | 4.87 ± 1.11 | 22% |
